# Non-equilibrium structural dynamics of supercoiled DNA plasmids exhibits asymmetrical relaxation

**DOI:** 10.1093/nar/gkac101

**Published:** 2022-02-21

**Authors:** Cynthia Shaheen, Cameron Hastie, Kimberly Metera, Shane Scott, Zhi Zhang, Sitong Chen, Gracia Gu, Lisa Weber, Brian Munsky, Fedor Kouzine, David Levens, Craig Benham, Sabrina Leslie

**Affiliations:** Department of Physics, McGill University, Montreal, QC H3A 2T8, Canada; Michael Smith Laboratories, University of British Columbia, BC V6T 1Z4, Canada; Department of Physics and Astronomy, University of British Columbia, BC V6T 1Z1, Canada; Department of Physics, McGill University, Montreal, QC H3A 2T8, Canada; Michael Smith Laboratories, University of British Columbia, BC V6T 1Z4, Canada; Department of Physics and Astronomy, University of British Columbia, BC V6T 1Z1, Canada; Department of Physics, McGill University, Montreal, QC H3A 2T8, Canada; Department of Physics, McGill University, Montreal, QC H3A 2T8, Canada; Institute of Materials Science, Kiel University, 24142 Kiel, Germany; Department of Physics, McGill University, Montreal, QC H3A 2T8, Canada; Department of Physics, McGill University, Montreal, QC H3A 2T8, Canada; Department of Physics, McGill University, Montreal, QC H3A 2T8, Canada; Department of Chemical and Biological Engineering and School of Biomedical Engineering, Colorado State University, Fort Collins, CO 80523, USA; Department of Chemical and Biological Engineering and School of Biomedical Engineering, Colorado State University, Fort Collins, CO 80523, USA; Center for Cancer Research, National Cancer Institute, Bethesda, MD 20892, USA; Center for Cancer Research, National Cancer Institute, Bethesda, MD 20892, USA; Genome Center, University of California Davis, Davis, CA 95616, USA; Department of Physics, McGill University, Montreal, QC H3A 2T8, Canada; Michael Smith Laboratories, University of British Columbia, BC V6T 1Z4, Canada; Department of Physics and Astronomy, University of British Columbia, BC V6T 1Z1, Canada

## Abstract

Many cellular processes occur out of equilibrium. This includes site-specific unwinding in supercoiled DNA, which may play an important role in gene regulation. Here, we use the Convex Lens-induced Confinement (CLiC) single-molecule microscopy platform to study these processes with high-throughput and without artificial constraints on molecular structures or interactions. We use two model DNA plasmid systems, pFLIP-FUSE and pUC19, to study the dynamics of supercoiling-induced secondary structural transitions after perturbations away from equilibrium. We find that structural transitions can be slow, leading to long-lived structural states whose kinetics depend on the duration and direction of perturbation. Our findings highlight the importance of out-of-equilibrium studies when characterizing the complex structural dynamics of DNA and understanding the mechanisms of gene regulation.

## INTRODUCTION

Life exists out-of-equilibrium. Cellular processes rely on transfers of energy and exchanges of materials to perform important biological functions. Studying the out-of-equilibrium dynamics of biological molecules and systems is necessary to understand many *in vivo* events. However, out-of-equilibrium studies pose a number of challenges. First, while there is a well-accepted framework to model biological systems at the molecular level using equilibrium statistical mechanics, a similar framework for out-of-equilibrium statistical mechanics does not exist (1). Furthermore, *in vitro* bulk measurements often focus exclusively on equilibrium states of molecules. In such bulk measurements, information about the time sequence of events, sub-populations of molecules and rare events is lost.

To tackle these obstacles, single-molecule techniques can be used to better study transitional structures, out-of-equilibrium states or stable sub-populations (2). However, many of these single-molecule techniques rely on tethering or otherwise constraining molecules, potentially altering the structure of the molecules they aim to observe. While these constraints can effectively be ignored for some systems, generally, tethering can restrict or alter the global structure of a molecule compared to the same molecule in free solution.

We have developed a single-molecule assay which builds on the Convex Lens-induced Confinement (CLiC) microscopy technique (3–5) and enables us to investigate out-of-equilibrium molecular dynamics in the absence of tethering. By confining molecules in solution within micrometer-scale wells, CLiC microscopy enables long observation times without the use of molecular tethers. This allows for structural studies of molecules without altering their global configuration or restricting states that may otherwise be present free in solution.

In previous work, we used this technique to study supercoiling-induced unwinding in plasmid DNA (4,5). We introduced a fluorescently labeled oligonucleotide complementary to a sequence-specific unwinding site that would only bind to that site if the site was in the single-stranded DNA conformation. As the size of the oligo probe (20–30 bases) was orders of magnitude smaller than a bound oligo-plasmid complex (approximately 2500 bp), bound complexes diffused far more slowly (approximately 100× slower) than free oligos in solution. The fraction of bound complexes in a given observation volume could then be counted by using CLiC microscopy. In this way an average 400 oligos trapped in micro-wells could be imaged simultaneously and through serial measurements, thousands of molecules can be probed.

In the present work, we extend this technique to probe the dynamics of the winding-unwinding transition under out-of-equilibrium conditions. We use temperature to shift the plasmids away from equilibrium, where heating the plasmids shifts the majority of them into an unwound state. After cooling to the experimental temperature, the DNA required time to form a double helix again. We sampled the plasmids at various time points after this disturbance, introducing the oligo immediately prior to imaging and measuring oligo binding over time. From the resulting binding curves we could estimate the rate of winding and unwinding, as well as the rate of oligo-plasmid binding.

We mathematically modeled the plasmid-probe interactions and the underlying structural transitions in order to extract kinetic information from our experimental data, and we quantified parametric uncertainties using Markov Chain Monte Carlo (MCMC) methods. We applied this technique to study the dynamics of structural transitions in supercoiled plasmid DNA and the effects of global DNA structure on the dynamics of such transitions.

To the best of our knowledge, no single-molecule experiments to date have examined the dynamics of supercoiling-induced transitions in DNA plasmids. While entire DNA plasmids have been studied in the past, these studies focused on atomic force microscopy (6), cryo-electron microscopy (7), or other techniques where the DNA is immobilized prior to imaging. Secondary structures in plasmids or genomes are typically studied in bulk through gel electrophoresis (8), with few studies focusing on the competition among secondary structures (9). Single-molecule studies of transitional dynamics, such as magnetic tweezer studies, have focused on the formation of a single secondary structure (10). Competitions among different structures was not evaluated. While these studies are informative, little information is available regarding the out-of-equilibrium dynamics of DNA unwinding sites in molecules containing other competing secondary structures, or how the presence of these structures impacts the unwinding and winding dynamics of the DNA.

In this paper we use CLiC microscopy to study the rate of the B-DNA unwinding transition in circular DNA plasmids. We estimate the state transition rates associated with DNA winding, DNA unwinding and oligo-plasmid binding for two plasmids, pFLIP-FUSE and pUC19, with different secondary structures. We use temperature to shift the proportion of wound and unwound plasmids away from the equilibrium state by heating or cooling the plasmids shortly before observation. Finally, we investigate the presence of other secondary structures in the plasmids and discuss the role of competition among structures on the dynamics of the winding/unwinding transition.

## MODEL SYSTEM AND BIOLOGICAL CONTEXT

### DNA superhelicity

In cells, DNA commonly is supercoiled, either over- or under-twisted. At least transient supercoiling accompanies all genetic transactions, especially where enzymatic machines translocate across stretches of DNA (11). The amount of supercoiling experienced by the DNA can be quantified by its supercoiling density (σ), the number of twists added to or removed from a relaxed molecule normalized by the size of that molecule. Conventionally, σ is positive when the molecule is over-wound relative to the relaxed state (i.e. has an excess of right-handed twists) and negative when it is under-wound. The torsional strain provided by this over- or under-twisting drives structural transitions between different secondary structures, both globally and at sequence-specific locations (12). These sequence-specific secondary structures include unwinding, Z-DNA formation and cruciform formation (13–16).

Of particular interest are the unwinding regions: AT-rich regions susceptible to stable melting at low temperatures when sufficiently negatively supercoiled. They facilitate the binding of transcription factors and RNA polymerases involved in the initiation of gene expression (8,17–19). For example, the *c-myc* proto-oncogene in the human genome is preceded by an unwinding region called the Far Upstream Sequence Element (FUSE). Under sufficient negative supercoiling, the FUSE region unwinds, allowing FUSE binding proteins (FuBPs) to bind, which regulates transcription of the *c-myc* gene (17,20).

The biophysics of supercoil-induced structural transitions have been theoretically modeled using equilibrium statistical mechanics. In 2015, Zhabinskaya and Benham published their DZCB-*trans* model (currently called *SIST* and available on the BitBucket website) (16). *SIST* uses Boltzmann statistics to analyze competitions among three types of alternate structures: unwinding, B-Z transitions, and cruciform extrusion. The model predicts the equilibrium probability of each type of transition occurring at base pair resolution in a supercoiled plasmid. Throughout this work, comparisons are made to predictions from the *SIST* model.

### Implications for gene regulation

Since each transition event localizes some of the negative supercoiling as undertwist, and the total amount of negative supercoiling is fixed, each transition in a plasmid diminishes the stresses felt by its other sites. This produces a global competition among all susceptible sites that may have regulatory implications. Changes in DNA supercoiling and interactions among susceptible sites can link the expression of multiple genes to one another. Since RNA polymerases generate transient supercoils as they travel along a DNA molecule, they can stimulate the opening or closing of nearby unwinding sites that act as promotor regions and transcription factor binding sites, linking the expression of neighboring genes (8,18,21,22). When multiple secondary structure-susceptible regions occur within the same topological domain, they compete for the available torsional strain. This can give rise to possibly complex interactions among them. For example, Z-DNA can act as a supercoiling ‘sink’, absorbing torsional strain that could otherwise drive unwinding and commence gene transcription (15). In some cases, supercoiling can coordinate gene expression throughout entire bacterial genomes, where increases or decreases in the expression of topoisomerase and gyrase can adjust global supercoiling levels to induce quick responses to changing environments (19), simultaneously activating or deactivating hundreds of genes (23,24).

DNA structure *in vivo* is constantly being perturbed, which makes the out-of-equilibrium dynamics of structural transitions increasingly important to understand. When DNA is perturbed away from equilibrium, the structures most likely to transition first are those with the fastest transition dynamics, as opposed to those with the lowest free energies. As the DNA relaxes back to equilibrium, it will eventually adopt the most energetically favorable configuration, but the complicated dynamics of such transitions has not been modeled to date. Detailed study of these subtle and complex super-structural interactions is made possible with single-molecule resolution using our non-tethered CLiC technique, which allows the DNA to freely explore all possible conformations.

### Model plasmids: pUC19 and pFLIP-FUSE

We investigated the dynamics of strand unwinding in two model plasmids. The first plasmid, pUC19, is a common cloning vector that contains two supercoil-induced unwinding regions (25). The most easily unwound region occurs upstream from the origin of replication, and the second region is associated with the ampicillin resistance gene. The region upstream from the origin of replication is our focus of interest, both in our previous work (4,5) and in this work.

The second plasmid, pFLIP-FUSE, was created by inserting the FUSE region of the *c-myc* oncogene into a cloning vector constructed from fragments of pUC19 and pEGFP-C1 (17). In this plasmid, the ampicillin resistance gene was replaced with a kanamycin resistance gene, which is not associated with a supercoil-induced unwinding region. Thus, the only unwinding region in the pFLIP-FUSE plasmid is the FUSE region itself.

### Reaction model

**Figure 1.**
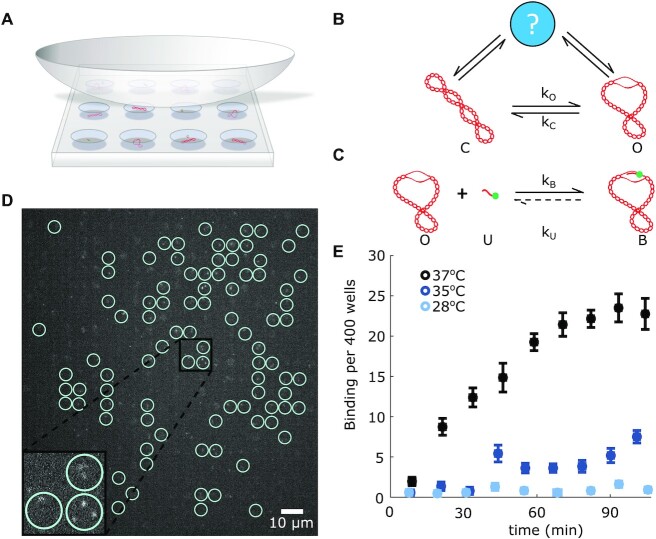
Oligo-binding assay for plasmid state determination. (**A**) Schematic of Convex Lens-induced Confinement (CLiC) microscopy. A flow cell is created by adhering two coverslips with double-sided tape. Micrometer-sized pits were etched into the bottom coverslip using lithographic techniques. The top coverslip is depressed with a convex lens to trap molecules in the pits for prolonged observation. For illustrative purposes, a mean value of one plasmid per pit is shown, though in experiments on average there are 23 plasmids and 0.82 oligos per pit. (**B**) The supercoiled plasmid is modeled as transitioning between an open *O* and closed state *C*. This is a simplification which assumes that the unwinding site is the only structure present in the plasmid. Estimates of the opening and closing rates are implicitly affected by the presence of other structures, indicated here by a question mark. (**C**) An oligonucleotide can bind to an open site to form a bound oligo-plasmid complex. Oligo unbinding was so slow that for the purposes of our experiments, this reaction was assumed to be irreversible. (**D**) Approximately 400 pits can be observed in a single field of view. Bound oligos are characterized by their slower diffusion relative to free oligos. Circles indicate pits containing bound oligos. The total number of bound oligos is counted in each video. Inset shows pits containing 0, 1, 2 or 3 bound oligos. (**E**) The number of bound oligo-plasmid complexes averaged over 10 videos as a function of time of the plasmid pFLIP-FUSE (σ = −0.10 ± 0.01) interacting with a 20 bp long oligonucleotide at a series of temperatures. Error bars represent the standard error of the mean. Plasmids were kept at 4°C prior to the start of the experiment, when they were mixed with oligo and brought to the experimental temperature.

We estimated kinetic rate constants by modelling the plasmid-oligo system as a series of molecular interactions, as shown in Figure 1B and C. First, the plasmid unwinding region can take on two states: open (O) and closed (C). Then the open region can interact with unbound oligos (U), forming bound oligo-plasmid complexes (B). Using the law of mass action, the following system of differential equations can be derived that describe this system:

(1) $$\begin{eqnarray*} \frac{\mathrm{d}[C]}{\mathrm{d}t} &=& k_{c}[O]-k_{o}[C] \nonumber \\ \frac{\mathrm{d}[O]}{\mathrm{d}t} &=& k_{o}[C]-k_{c}[O]-k_{b}[O][U] \nonumber \\ \frac{\mathrm{d}[U]}{\mathrm{d}t} &=& -k_{b}[O][U] \nonumber \\ \frac{\mathrm{d}[B]}{\mathrm{d}t} &=& k_{b}[O][U] \end{eqnarray*}$$

Here, [O], [C], [U] and [B] are the concentration of molecules in the states O, C, U and B, respectively (see Figure 1B). *k*_*c*_ is the chemical rate constant of the unwinding site closing, *k*_*o*_ is the chemical rate constant of the unwinding site opening, and *k*_*b*_ is the chemical rate constant of the plasmid and oligo binding to form a plasmid-oligo complex. *k*_*b*_ encompasses both the rate of the oligo and plasmid entering the same space, and the rate of hybridization once collision occurs. The rate of the oligo dissociating from the plasmid was found to be much longer than the timescale of our experiments and is assumed to be negligible in our model (4).

For all experiments, the total concentration of oligonucleotides in the experiment was 0.752 nM (on average, 0.82 oligos per well). In pUC19 experiments, the total concentration of plasmids in any state (O, C or B, excluding nicked plasmids), *P*_*o*_, was 21.1 nM (on average, 23 plasmids per well). In pFLIP-FUSE experiments, the total concentration of plasmids, *P*_*o*_, was 11.9 nM (on average, 13 plasmids per well). These concentrations were chosen to obtain appreciable binding under the experimental conditions.

These differential equations were fit to experimental data using an MCMC sampler (Metropolis algorithm, see Supplementary Information for more information) to estimate each of the chemical rate constants and the initial fraction of open plasmids, [*O*](*t* = 0).

The experiments measured the number of oligos bound to plasmids as a function of time, [*B*](*t*). For each heat treatment, several concentration curves were measured, differing only by an incubation period (*t*_inc_) after the perturbation and prior to the addition of oligo. According to our model, the concentration of unwound plasmids at the times when oligos were added, [*O*](*t*_inc_), is:

(2) $$\begin{eqnarray*} [O](t_{\text{inc}}) &=& \frac{P_{o}k_{o}}{k_{o}+k_{c}}+\left[[O](t_{inc}=0)-\frac{P_{o}k_{o}}{k_{o}+k_{c}}\right] \nonumber \\ &&\times\, e^{-(k_{o}+k_{c})t_{\text{inc}}} \end{eqnarray*}$$

where [*O*](*t*_inc_ = 0) is the concentration of unwound plasmids when the oligo was added to the curve with the smallest *t*_inc_. To estimate the fraction of unwound plasmids at equilibrium (without oligos present), we took the limit of Equation (2) as *t*_*inc*_ approaches infinity.

## MATERIALS AND METHODS

### Plasmid preparation

#### Plasmid growth and purification

Plasmids were extracted from DH5α *Escherichia coli* grown in lab, by using a QIAfilter plasmid midi kit (QIAGEN). The plasmids were supercoiled by reacting them with calf-thymus topoisomerase 1B (Life Technologies) in the presence of various concentrations of ethidium bromide (26) and purified using a QIAquick mini PCR purification kit (QIAGEN). DNA concentration was determined from the OD_260_ of the eluted DNA. Quality and supercoiling density of the plasmids was verified through gel electrophoresis (Supplementary Figure S1) (26). This method produced negatively supercoiled plasmids (pFLIP-FUSE or pUC19) with a Gaussian distribution of supercoiling densities about a mean value. Values of supercoiling densities used in this paper are followed by the standard deviation of this Gaussian distribution to indicate the spread of supercoils in the sample. Plasmids were stored in 10 mM Tris (pH 8.0) at 4°C until use.

#### Sample preparation for microscopy

Unless otherwise noted, each sample consisted of 21.1 nM plasmid DNA, 752 pM oligonucleotide probe (20 bases long for pFLIP-FUSE, 30 bases long for pUC19, see Supplementary Information for sequence and labeling information) labeled with Cy3b via an NHS ester bond, 25 mM HEPES, 10 mM NaCl, 12 mM Tris-HCl (pH 8.0), 2.5 mM protocatechuic acid (PCA), 50 nM protocatechuate 3,4-dioxygenase (PCD), 1 mM KCl, 20 µM EDTA and 1 % glycerol. PCA and PCD are a substrate enzyme pair that remove oxygen from solution, reducing the amount of photobleaching. The KCl, EDTA and glycerol were from the storage solution for the PCD. For any samples with a pre-experiment temperture treatment, all components except the probe oligo, PCA, PCD and associated storage buffer were mixed before treatment. Oligo, PCA and PCD were added after treatment, immediately prior to microscopy.

Samples were subjected to one of two heat treatments prior to visualization on the microscope. All samples were stored at 4°C (which favours no unwinding) in 10 mM Tris-HCl buffer (pH 8.0) prior to experiments. For heat treatments, they were mixed with the buffer described above, without the oligo, PCA, PCD and associated storage buffer. Samples that approached equilibrium from a closed state (‘chilled’ samples) were heated to 37°C with a Biorad C1000 Touch Thermal Cycler and held at that temperature for the desired incubation time (*t*_inc_). Samples that approached equilibrium from an open state (‘heated’ samples) were heated with a Biorad C1000 Touch Thermal Cycler to 95°C for one minute unless otherwise noted, cooled at a rate of 5°C/min and held at 37°C for the desired incubation time (*t*_inc_). Samples were removed from the thermocycler immediately prior to the start of the oligo-binding reaction. Oligos, PCA and PCD were added to start the reaction as the samples were transferred to the microscope, which was heated to 37°C and held there. In experiments labelled with ‘no heating’, plasmids were mixed with oligo, PCA and PCD immediately after removal from 4°C, then imaged on the microscope at 37°C with no initial heating step.

### Microscopy

#### Convex lens-induced confinement

Convex lens-induced confinement (3,5,27) was used in all microscopy experiments to confine molecules in 3 µm wide, 500 nm deep pits. Pits were etched into D263 cover glass using UV lithography as described in prior work (4). Flow cells were constructed by affixing the pitted glass to a second cover glass that had two inlet ports drilled through it, using double sided tape as a spacer. Flow cells were secured in the microfluidic chuck of a CLiC device and mounted on a Nikon Eclipse TI-E inverted microscopy with a CFI Apo TIRF 100× objective. Sample was flowed into the flow cell through the inlet ports, applying a positive pressure to facilitate any necessary buffer exchange. Samples in the flow cell were maintained at 37°C using the heating system detailed in (4).

#### Measuring concentration curve

To measure a concentration curve, sample was added to the flow cell, using positive pressure to displace a blank buffer. The CLiC lens was lowered, depressing the top coverslip of the flow cell and confining molecules in wells (Figure 1A). Wells were illuminated with a 532 nm Sapphire laser (Coherent), and a 50-ms exposure, 100-frame video was taken using an EMCCD camera (Andor iXon Ultra 888 EMCCD camera). The CLiC lens was lifted to release the sample. This process was repeated once per minute until ninety videos were acquired. In each video, the bound oligo-plasmid complexes were distinguishable from the free oligos by a large change in diffusion. In one frame at 50 ms exposure, the bound complex was a localized diffraction limited spot, while the free oligo was a diffuse ‘smear’. This is illustrated in Figure 1 D, where one frame from a representative video is shown with bound molecules circled for illustrative purposes.

### Analysis

Well locations in each video were automatically detected (see Supplementary Information for more information) and bound oligo-plasmid complexes were identified using the supervised machine learning algorithm described in (4). All counts were verified by human inspection. Since the wide-field laser beam had a Gaussian beam profile, wells toward the edges of the beam were too dark to accurately measure and excluded from analysis. Dark wells were experimentally determined to be wells that lay >0.55 standard deviations away from the peak of the Gaussian beam profile (see Supplementary Information for more information). All counts of molecules were scaled to be per 400 wells, to compensate for differences in the number of excluded pits from data set to data set. Data were averaged over every ten videos before displaying on graphs. A typical concentration vs time graph is shown in Figure 1E. In this curve, pFLIP-FUSE was mixed with oligo and imaged on the microscope at different temperatures, showing the relationship between temperature and binding. Where applicable, fits on graphs use the reaction model outlined in Equation (1). Fit parameters and uncertainties were estimated using MCMC sampling (Metropolis-Hastings method, see Supplementary Information and *Reaction Model* section for more information).

### Potassium permanganate footprinting

Secondary structures in DNA were detected and mapped using potassium permanganate footprinting analysis (28). Certain DNA structures, such as site-unwinding, cruciforms and Z-DNA, have single-stranded DNA regions. The entire unwinding region is single-stranded when in its unwound state, cruciforms create single-stranded bases at the B-cruciform junction and at the apex of the cruciform, and the B-Z junctions of Z-DNA contain one single-stranded base each (29). Thus, detecting the presence and locations of single-stranded DNA indicates the existence and positions of these secondary structures.

Potassium permanganate oxidizes single-stranded thymine bases, preventing them from closing. Here we probed DNA for alternate structures by treating it with potassium permanganate, linearizing with a restriction endonuclease, and cutting at single-stranded regions with an S1 nuclease. To perform this permanganate footprinting, 1 µg of DNA was prepared as it would be prior to a microscopy experiment (including pre-experiment heat treatments). Oligo, PCA and PCD, and the associated storage buffer were not used in the footprinting, to mimic the initial conditions of the binding curves. Because HEPES inhibits the potassium permanganate reaction, we substituted Tris-HCl for HEPES in the experimental buffer, bringing the final Tris concentration to 20 mM. On a heat block at 37°C, samples were treated with 7 mM of potassium permanganate for one minute to oxidize any single-stranded thymine bases. The permanganate was inactivated with β-mercaptoethanol and the DNA was purified. All DNA purifications were conducted using QIAquick mini PCR purification kit (QIAGEN), following the manufacturer’s instructions. Permanganate-modified plasmids were linearized with a restriction endonuclease. Purified linear DNAs were treated with S1 nuclease (NEB), following the manufacturer’s instructions. The restriction endonuclease varied by sample, using HindIII for pUC19 and NcoI-HF for pFLIP-FUSE (all enzymes from NEB). After a final purification eluted with 10 µl of 10 mM Tris-HCl (pH 8.0), samples were analyzed by electrophoresis on a 1% agarose gel. Gels were stained with SybrSafe and imaged.

## RESULTS

### Unwinding and winding transitions in pFLIP-FUSE

As with pUC19 (4), site-unwinding in pFLIP-FUSE increases with temperature (Figure 1E), in agreement with theoretical predictions (16) and bulk experiments with the pFLIP-FUSE plasmid (17). The samples used in Figure 1E were kept at 4°C until immediately prior to the experiment when they were mixed with the oligo and heated to the experimental temperature. Since site-unwinding is temperature dependent, we used temperature to induce large-scale unwinding and perturb plasmids away from an equilibrium partition of wound and unwound plasmids. While unwinding is increased in the plasmids through heating, after cooling only a minority of the total plasmids demonstrate unwinding. We added fluorescently labeled oligos to the solution at various times after perturbation and measured oligo binding over time to infer the number of unwound plasmids. Using this approach, we measured how long it took the plasmids to reach equilibrium at 37°C after a perturbation to a cooled state 4°C and also to a heated state 95°C.

**Figure 2.**
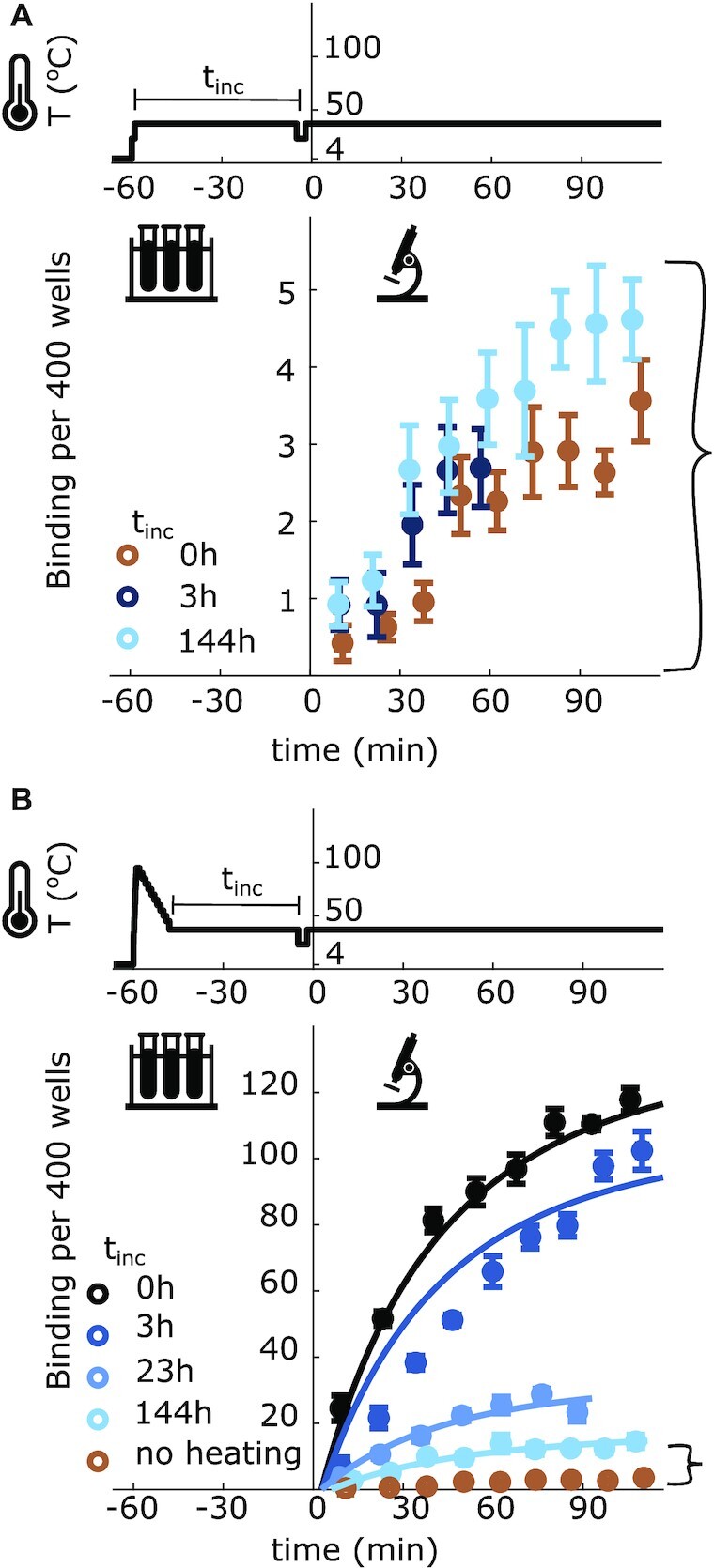
The number of fluorescent oligos bound to pFLIP-FUSE (σ = −0.062 ± 0.006) molecules per 400 pits, averaged over 10 videos, as a function of pre-experiment temperature treatment. (**A**) pFLIP-FUSE plasmids were heated from 4 to 37°C and held at this temperature for either 0, 3 or 144 h prior to the addition of the oligo and oxygen scavengers at *t* = 0 s. (**B**) Plasmids were heated to 95°C for 1 min and cooled to 37°C at a rate of 5°C/min to prevent mismatching. Plasmids were then held at 37°C for either 0, 3, 23 or 144 h, prior to the addition of the oligo and oxygen scavengers at *t* = 0 min. The ‘no heating’ curve is the 0 h curve from the graph in (A) and is included for scale. Brackets indicate areas of the same scale between (A) and (B). Fits are generated using the parameters in Table 1. All error bars represent the standard error of the mean. Top traces indicate the temperature of the plasmids. The decrease to room temperature just before time 0 indicates when the sample was transferred from the thermal cycler to the microscope.

The pFLIP-FUSE plasmids (σ = −0.062 ± 0.006) were stored at 4°C, a temperature at which site unwinding is improbable. We then brought the plasmids to 37°C and added the oligo after different incubation times to measure the presence of unwound plasmid. There was no observable change in the plasmid-oligo binding rate with incubation time as shown in Figure 2A. Since all other solution, sample, and temperature conditions were identical, this suggests that the partition of wound and unwound plasmids reached equilibrium very quickly after being heated to 37°C, or that the opening rate was sufficiently slow that no change in binding was observed with increasing incubation time. This same trend was observed with pUC19 (Supplementary Figure S7).

The plasmids behaved differently when heated to 95°C and then brought to 37°C. The plasmids were heated to 95°C for 1 min before being cooled at a rate of 5°C/min until they reached 37°C (Figure 2B). We then held the plasmids at 37°C for various lengths of time before adding the complementary oligo. When we added the oligo immediately after cooling, a large amount of oligo-plasmid binding was observed, suggesting a high number of unwound plasmids. The amount of binding decreased as the time between cooling and adding the oligo increased. No such increase was observed when an oligo complementary to another site on the plasmid was used (Supplementary Information, Supplementary Figure S5), demonstrating that the slow reannealing was specific to the unwinding site and that the rate limiting step is not large scale melting of the entire plasmid.

We used MCMC sampling (Metropolis algorithm) to estimate the initial and transient concentrations of wound and unwound plasmids, as well as the chemical rate constants associated with plasmid winding, plasmid unwinding and oligo-plasmid binding (see Equation 1) over the course of the experiment. All parameters were free to change during parameter estimation. The estimated parameters are summarized in Table 1, and their posterior distributions are shown in Supplementary Figure S2. Joint parameter uncertainties are summarized in the Supplementary Information in Supplementary Figures S3 and S4. The concentration of open unwinding sites at equilibrium (without oligos present) was calculated from the rates of opening and closing by taking the limit of Equation (2) as *t*_*inc*_ approaches infinity. As expected, the rate of oligo binding (*k*_*b*_) dominates the reaction. The rates of unwinding and winding are small at the studied conditions, leading to slow relaxation back to equilibrium after the plasmids are heated and then cooled to 37°C (Figure 2B). Also as predicted, at the given conditions the winding rate is higher than the unwinding rate, leading to a low proportion of unwound plasmids at equilibrium. From the rates, we calculated that at equilibrium $0.29 \pm 0.01\%$ of plasmids were unwound. Using the DZCB-*trans* algorithm (16), the probability of plasmid unwinding at these conditions is predicted to be 0.47%.

**Table 1.** Chemical rate constants of site-winding, site-unwinding, and oligo-plasmid binding, and equilibrium partitions of wound and unwound plasmids in pFLIP-FUSE (σ = −0.062 ± 0.006) and pUC19 (σ = −0.052 ± 0.006) plasmids at 37°C after heating to 95°C for 1 min (pFLIP-FUSE) or 60 min (pUC19), 22.5 mM ionic concentration. Values were estimated through fitting oligo-plasmid binding curves using MCMC methods. Uncertainties are the 68% confidence interval of the estimated parameter distribution

	pFLIP-FUSE	pUC19
*k*_*o*_ (s^-1^)	(7.0 ± 0.1) × 10^−8^	(6 ± 1) × 10^−8^
*k*_*c*_ (s^-1^)	(2.4 ± 0.1) × 10^−5^	(7.6 ± 0.6) × 10^−5^
*k*_*b*_ (M^-1^s^-1^)	(5.5 ± 0.9) × 10^5^	(2.5 ± 0.7) × 10^5^
[*O*](0)/*P*_*o*_	(2.6 ± 0.2) × 10^−2^	(1.2 ± 0.3) × 10^−2^
[*O*](∞)/*P*_*o*_	(2.9 ± 0.1) × 10^−3^	(7.8 ± 0.7) × 10^−4^

From these measured rates, we can see that the amount of unwound plasmids in Figure 2B has returned to equilibrium. However, should these rates apply to the conditions presented in Figure 2A, which starts from a condition where all plasmids are closed, we would expect the equilibrium partition of wound and unwound plasmids (0.29% of plasmids being open, as stated in Table 1) to be reached well before 144 h after heating. However, less binding than expected is observed in the chilled samples after incubation (Figure 2A and the brown curve in Figure 2B). Likewise, we would expect to observe less binding at shorter incubation times (see Supplementary Figure S11 for the simulated curves using the rates measured from Figure 2B).

This asymmetry in the binding rates, where there is a mismatch between the measured opening and closing rates after heating or cooling, in combination with our gel measurements, suggests that other secondary structures present in the plasmids may play an important role in determining the opening and closing rates of the unwinding site. Most notably, the amount of binding in Figure 2A did not converge with the amount of binding observed at equilibrium in Figure 2B, suggesting that there is a long-lived state present in either the cooled or heated sample slowing the transition to equilibrium.

### Unwinding and winding transitions in pUC19

**Figure 3.**
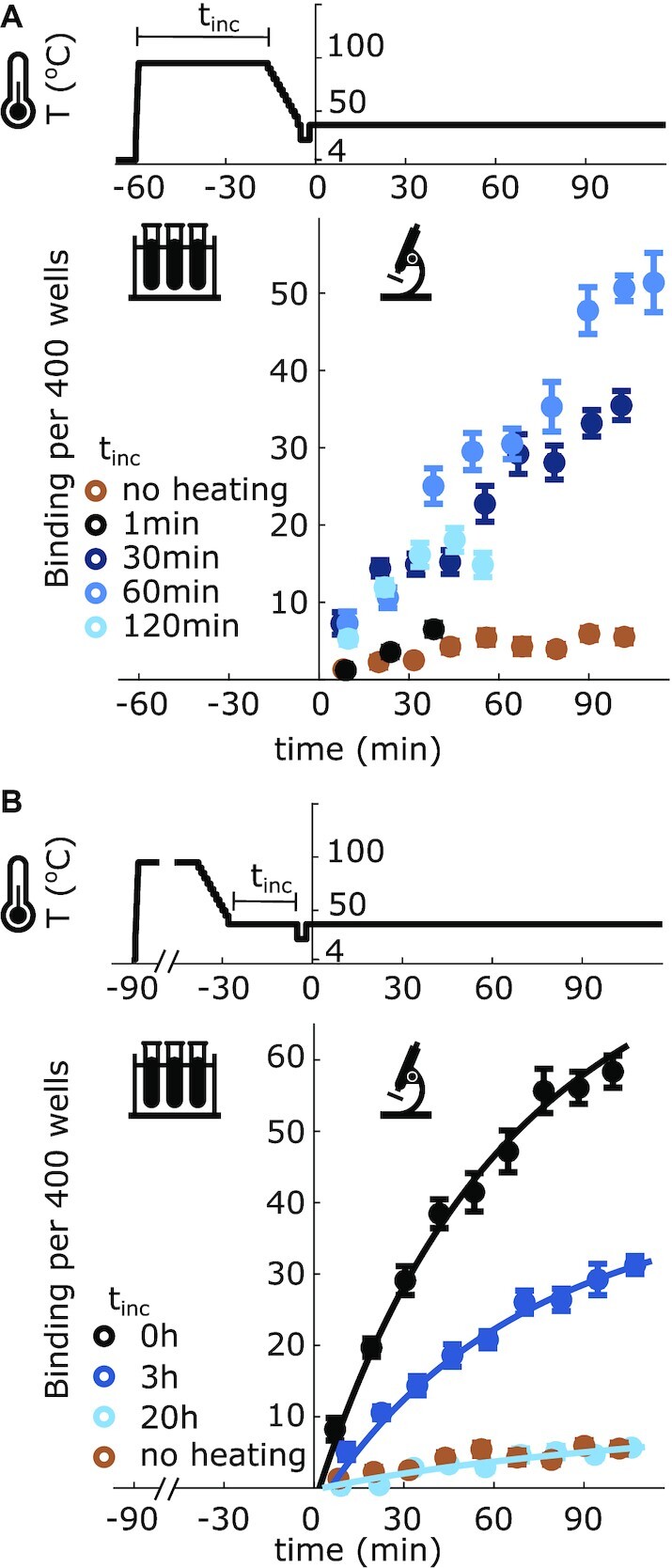
The number of fluorescent oligonucleotides bound to pUC19 (σ = −0.054 ± 0.006) molecules per 400 pits, averaged over 10 videos, with respect to different pre-experiment temperature treatments. (**A**) pUC19 plasmids were held at 95°C for either 1, 30, 60 or 120 min before being cooled to 37°C at a rate of 5°C/min prior to the addition of the oligo and oxygen scavengers at *t* = 0 min. (**B**) Plasmids were heated to 95°C for 60 min and cooled to 37°C at a rate of 5°C/min. Plasmids were then held at 37°C for either 0, 3 or 20 h, prior to the addition of the oligo and oxygen scavengers at *t* = 0 min. The 'no heating' curve on both graphs is pUC19 that had been heated directly to 37°C prior to the addition of the oligo. Curves are fitted using the parameters in Table 1. All error bars represent the standard error of the mean. Top traces indicate the temperature of the plasmids. The decrease to room temperature just before time 0 indicates when the sample was transferred from the thermal cycler to the microscope.

We subjected supercoiled pUC19 plasmids (σ = −0.054 ± 0.006) to the same experimental conditions we used for pFLIP-FUSE. Heating pUC19 to 95°C for 1 min did not increase unwinding (Figure 3A). This was unexpected, as 1 min was sufficient to increase the unwinding in pFLIP-FUSE plasmids, regardless of supercoiling density (Supplementary Figure S6). However, pUC19 needed to be heated longer to elicit a shift in unwinding after cooling (Figure 3A). The increase in unwinding stabilized for heating times longer than 30 min.

When pUC19 was heated to 95°C for 1 h and then cooled to 37°C the relaxation back to equilibrium was faster than observed with pFLIP-FUSE (Figure 3B). As with pFLIP-FUSE, the amount of oligo binding increased for plasmids which were heated and decreased with longer incubation times. With pUC19 (σ = −0.054 ± 0.006), 20 h of incubation was sufficient for the plasmids to return to their equilibrium partition of wound and unwound sites.

We estimated the chemical rate constants and initial concentration of unwound plasmid using MCMC methods. The fitted rate constants are summarized in Table 1. As with pFLIP-FUSE, the oligo binding rate dominates the reaction. As above, we calculated the equilibrium concentration of unwound plasmids without oligos present from the fitted rate constants. At equilibrium $0.078 \pm 0.007\%$ of plasmids had an open unwinding site. Consistent with our prior work (4), the experimental amount of unwinding was less than predicted ($0.33\%$ at *T* = 37°C, [*Na*^+^] = 22.5 mM and σ = −0.054, calculated with DZCB-*trans* algorithm (16)).

### Secondary structures globally present in plasmids

We conducted potassium permanganate footprinting (28) to examine the secondary structures present in these plasmids. First, we prepared supercoiled plasmids in experimental buffer and subjected them to the same temperature treatments used for microscopy. We either heated them to 95°C for 1 min and cooled them to 37°C or heated them directly to 37°C. We treated the plasmids with potassium permanganate, linearized them with a restriction enzyme, then treated the samples with an S1 nuclease. We ran the fragments on an agarose gel and mapped the cut locations based on fragment length.

**Figure 4.**
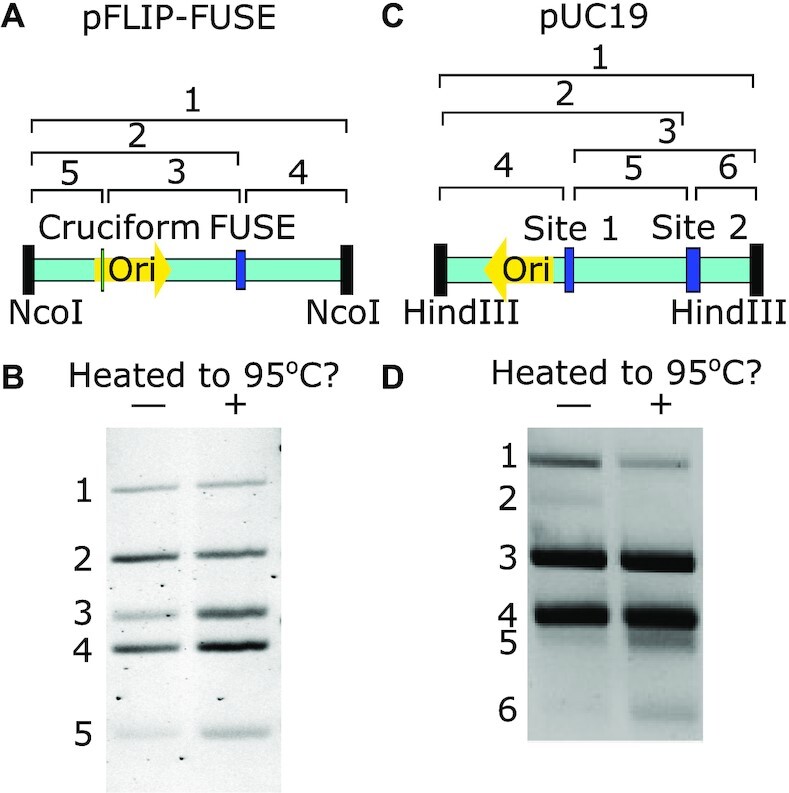
Secondary structures present in the plasmids pFLIP-FUSE (σ = −0.10 ± 0.01, (**A** and **B**)) and pUC19 (σ = −0.097 ± 0.009, (**C** and **D**)). (A) Schematic illustrating the location of the FUSE region and a cruciform region in pFLIP-FUSE with respect to the NcoI-HF cut site and origin of replication (Ori). (C) Schematic illustrating the location of the two unwinding sites in pUC19 with respect to the HindIII cut site and origin of replication (Ori). (**B** and **D**) Potassium permanganate footprinting analysis illustrating secondary structures present in pFLIP-FUSE (B) or pUC19 (D) when directly heated to 37°C (left lane, -) or heated to 95°C and cooled to 37°C (right lane, +). After heating, plasmids were treated with potassium permanganate, then cut with either NcoI-HF (pFLIP-FUSE) or HindIII (pUC19) followd by an S1 nuclease treatment. The S1 nuclease cut the plasmids at the structures indicated in (A) and (C), if they were present.

Supercoiled pFLIP-FUSE plasmids (σ = −0.10 ± 0.01) heated to 37°C produced five fragments (Figure 4B), suggesting that there were two cut sites, corresponding to the FUSE unwinding region and a cruciform-susceptible region (see Supplementary Information Supplementary Figures S8 and S9 for mapping). At σ = −0.10 ± 0.01, the observed bands correspond to plasmids that had no secondary structures, plasmids with just the FUSE unwinding region open, and plasmids where both the FUSE region was open and the cruciform was extruded.

Plasmids brought directly to 37°C from a cooled state of 4°C (left lane of Figure 4B) exhibited less unwinding and less cruciform extrusion than plasmids heated to 95°C and cooled to 37°C (right lane of Figure 4B). This again shows a slow relaxation back to equilibrium after a perturbation.

Interestingly, regardless of heat treatment, plasmids were not observed in a state where the cruciform was extruded while the FUSE unwinding region remained closed. However, such a state was observed in less supercoiled plasmids (σ = −0.058 ± 0.007) (Supplementary Information Supplementary Figure S10), demonstrating that the order of structural transitions has a dependence on supercoiling. Additionally, at lower superhelicities (σ = −0.050 ± 0.007) we observed that the unwinding region is the first region to open with increasing temperature or decreasing ionic concentration (Supplementary Figure S14).

In pUC19 (σ = −0.097 ± 0.009), we observed two cut sites, corresponding to the locations of the two known unwinding regions (see Supplementary Information Supplementary Figure S9 for mapping) (Figure 4D). As with pFLIP-FUSE, after heating to 95°C and subsequently cooling to 37°C we saw an increase in plasmids with both unwinding sites open. In samples heated directly to 37°C from 4°C we observed plasmids where the secondary unwinding site had opened while the first remained closed (Figure 4D, band 2). However, in samples heated to 95°C and cooled to 37°C, this band was no longer present, suggesting that after this heat treatment the secondary unwinding site was only open in plasmids that also contained an open primary unwinding site. For further discussion and computational predictions of secondary structures in both pUC19 and pFLIP-FUSE, refer to Supplementary Information.

## DISCUSSION

In this work, we investigated the out-of-equilibrium dynamics of unwinding transitions in supercoiled DNA. We compared the dynamics between two different plasmids, pFLIP-FUSE and pUC19. As pFLIP-FUSE and pUC19 have different sequences, and thus different propensities for forming secondary structures, we compared and contrasted the estimated opening and closing rates of their unwinding sites to better understand the role of global structure on the dynamics of supercoil-induced site melting and structural transitions in general.

Interestingly, the rate of the unwinding site transition was asymmetric, dependent on whether the plasmids were heated or chilled prior to reaching physiological temperatures. After the pUC19 plasmids were heated before being brought to physiological temperature, their unwinding sites took a long time to return to their equilibrium concentrations compared with samples that were initially cooled. This timescale was on the order of hours (Figure 3B). The kinetic rates, predicted using the data from the heated plasmids, cannot be extrapolated to describe the behavior of the chilled plasmids (Supplementary Figure S11). More specifically, pFLIP-FUSE plasmids transferred to 37°C from 4°C did not reach the levels of binding predicted at equilibrium from those kinetic rates, even after a week of incubation. After 144 h of incubation, we observed no change in the level of binding for the chilled plasmids, when the estimated rates from the heated plasmids predict an increase. Similarly, with the rates measured from the heated plasmids we would expect chilled pUC19 to take approximately 10 h to reach equilibrium, but the chilled samples reach an equilibrium level of binding almost immediately (Supplementary Figure S11). In both cases, this suggests that the rate of site opening and closing depends on whether the plasmids approached equilibrium from much higher or much lower temperatures. This shift in the rate constants based on preparation suggests that multiple secondary structures play a role in the dynamics of unwinding of the examined sites. More non-B-DNA structures are expected to occur at higher temperatures, which could explain the slower relaxation dynamics in pUC19 when approaching equilibrium from high temperatures. In pFLIP-FUSE, the amount of binding observed in chilled samples did not converge with the amount observed in the heated sample, even after each was incubated for a week. In fact, in both scenarios, the amount of binding appears to have stabilized, suggesting that there is some energy barrier, most likely caused by the presence of secondary structures, that is slowing down the winding-unwinding transition.

Hysteresis in torsional-induced structural transitions has been observed in the past. King *et al.* observed hysteresis in ‘strand-unpeeling’ (ssDNA originating at ends or nicks in the DNA) when torsion was applied to DNA using optical tweezers (30). They also observed competition among three structural transitions, unwinding, unpeeling and a transition to S-DNA, where different structures were preferred under different environmental conditions. Though they did not observe a hysteresis in unwinding under changing torsion, such hysteresis was observed by Zhang *et al.* after a change in torque on the molecule (31). This supports our observation of hysteresis in the unwinding transition after a temperature change.

The differences in relaxation rates between pUC19 and pFLIP-FUSE support the hypothesis that secondary structures are affecting the winding-unwinding transition rates. Under physiological conditions, the primary unwinding site in pUC19 is less stable than that in pFLIP-FUSE. The pUC19 unwinding site is longer and has a lower % GC content, leading to a lower melting temperature than that in pFLIP-FUSE, both with and without supercoiling. When the plasmids were heated from a cooled state, this lower melting temperature was observed, as pUC19 exhibited more oligo-binding than pFLIP-FUSE under similar conditions (Figures 2A and 3A, brown curves). Despite pUC19 containing an unwinding site with a lower melting temperature, it required longer incubation times at 95°C to exhibit significant melting than pFLIP-FUSE did under similar conditions. Additionally, the maximum oligo-binding observed in pUC19 after heating and cooling was less than that observed in pFLIP-FUSE, and the levels of binding reached equilibrium faster with pUC19 (Figures 2B and 3B). Overall, the unwinding region in pUC19 exhibited a higher rate of closing. This was surprising, since the unwinding region in pUC19 was predicted to melt more easily. Thus, the presence of other non-B-DNA secondary structures, which are expected at higher temperatures, is likely altering the transition dynamics of the unwinding region as it closes after heating.

The permanganate footprinting provides further evidence that global competitions affect the dynamics of secondary structures *in vitro* (Figure 4). Two single-stranded secondary structures were detected in both pUC19 and pFLIP-FUSE. In pUC19, the primary and secondary unwinding sites were detected, while in pFLIP-FUSE, the FUSE unwinding site and a cruciform were detected. In pFLIP-FUSE plasmids under large negative superhelicities (σ = −0.10 ± 0.01), the cruciform was only observed in plasmids that also contained an open unwinding region, suggesting an ordering to the structural transitions. This reflects hypotheses made on a similar Z-DNA containing system (15), where the energetically favored structure was predicted to form first, and other structures only formed if there was sufficient excess energy. Our observations suggest a similar mechanism: since the unwinding site is energetically favored at higher temperatures and negative supercoiling, it forms first, while the cruciform only forms if there is sufficient excess energy (Supplementary Figure S12). At lower supercoiling density (σ = -0.058 ± 0.007), the cruciform was present in plasmids which did not contain an open unwinding site because the cruciform is energetically favored at these conditions (Supplementary Information Supplementary Figures S10 and S12). We observed similar ordering with pUC19 upon perturbing the system to a heated or cooled state. However, when we brought plasmids to physiological temperatures from a cooled state, we observed populations where the secondary unwinding site was open in the absence of an open primary unwinding site. This is consistent with theoretical predictions that suggest that at high temperatures both sites would be open at the same time (Supplementary Figure S13). Overall, we observed competition and ordering in the formation of secondary structures, indicating that global correlations between structures affect transition dynamics out-of-equilibrium.

The footprinting analyses indicate that the ordering of secondary structure formation is dependent on whether molecules were heated or cooled prior to reaching 37°C. This provides insight into the mechanism causing a discrepancy in the opening and closing rates of the plasmid unwinding sites measured on the microscope. Alternative transitional pathways of all secondary structures, each associated with their own transition rates, can cause the dynamics of transition to be complicated.

Our measurements suggest that the hysteresis observed in the relaxation toward equilibrium of DNA structures is due to competition among all alternate structures within a topological domain. The presence of long-lived alternate structures may stabilize the unwinding site and keep the unwinding site open for longer than would otherwise be expected. This creates a delay between structural perturbations and the relaxation back to equilibrium, a property which may have interesting consequences specific to DNA function.

While the conditions we present in this paper are not physiological, we provide evidence that the competition among DNA structures can lead to a hysteresis in the rate of DNA structural transitions. The observed winding and unwinding rates are slower than would be expected if there were no competition among secondary structures. Additionally, the rates were asymmetrical: the transition rates of the heated sample cannot be applied to the transition rates of the chilled sample (Supplementary Figure S11).

Since DNA is constantly perturbed away from equilibrium *in vivo*, this hysteresis could be utilized for cellular processes. There are several instances in biology where DNA has ‘memory’, ranging from epigenetic markers (32) to bookmarks that occur during mitosis (33,34). Most research into these phenomena focuses on protein–DNA interactions or other physical markers. However, some evidence suggests that a perturbation to the DNA is necessary before bookmarking with proteins can occur (33). Despite this fact, the structural implications for DNA when such a perturbation occurs remain largely unexplored. Further study is necessary to see if the hysteresis observed in this study happens under such conditions. Should they be present, the study of out-of-equilibrium structural dynamics of DNA should help in the discovery of models for DNA memory by providing a mechanism of ‘short-term memory’, where a structural perturbation can allow for increased protein–DNA interactions for a transient time after that perturbation. This is similar to how the modification of the DNA with a protein or marker can create a ‘long term memory’ for the DNA (32).

Competitions among secondary structures and their effects on transition dynamics play important roles in gene regulation. The activities of some genes are controlled in part by a supercoil-induced unwinding site upstream from the gene, which allows for protein binding when it is open (8,17,18). The expression of neighboring genes can be linked through supercoils generated by RNA polymerases (18). Also, the expression of genes across the entire bacterial genome can be linked, either by altering the transcription levels of topoisomerases and gyrases (19), or by changes in gyrase activity induced by altered ATP concentrations (24). This latter method of gene regulation is so important that in a long term evolution experiment, 10 of 12 bacterial colonies grown in a minimal media environment evolved to be more negatively supercoiled than the ancestral colony (35). Our results suggest that the timescales of structural transitions after a large-scale change in environment are non-trivial and depend on the global structure of a topological domain, not just the structure of a specific unwinding site. These timescales could lead to vastly different dynamics for various structural transitions after perturbation or environmental change, adding far more control of which genes are expressed and in what order.

This work opens the door to future studies involving the dynamics of supercoil-induced structural transitions in DNA. We presented a model system to establish our ability to probe these dynamics and showed that the dynamics are dependent on competitions among secondary structures. In future work, we will investigate these competitions more explicitly, comparing the structural transition rates when a variety of competing structures are present. We will also extend beyond perturbations of temperature to explore perturbations to supercoiling caused by enzyme activity.

Overall, the hysteresis observed in DNA structural transitions after heating or cooling illustrates the importance of investigating the dynamics of out-of-equilibrium transitions in the native biological context, where competing structures are unhindered. While some theoretical models of the behaviour of supercoil-induced structures at equilibrium exist (15,16,36), extending these models to predict out-of-equilibrium dynamics is a new pursuit (37).

## CONCLUSION

We have presented a new method of studying out-of-equilibrium structural dynamics in supercoiled DNA. Using CLiC microscopy, we studied the dynamics of these transitions without tethering the molecules, allowing them to explore their natural configurations. By perturbing the system from equilibrium using temperature, we studied the out-of-equilibrium dynamics of the system as it relaxed back to equilibrium. We also estimated chemical rate constants of two step reactions, consisting of a state change and two molecules binding. This method can be expanded to many applications, to characterize structure-function interactions and out-of-equilibrium dynamics.

## DATA AVAILABILITY

All data in this study are available upon request through contacting the corresponding author.

## Supplementary Material

gkac101_Supplemental_File

## References

[1] Chou T. , Mallick K., Zia R. Non-equilibrium statistical mechanics: from a paradigmatic model to biological transport. Reports on progress in physics. 2011; 74:116601.

[2] Miller H. , Zhou Z., Shepherd J., Wollman A.J.M., Leake M.C. Single-molecule techniques in biophysics: a review of the progress in methods and applications. Rep. Prog. Phys. 2017; 81:024601. 10.1088/1361-6633/aa8a02 28869217

[3] Leslie S.R. , Fields A.P., Cohen A.E. Convex lens-induced confinement for imaging single molecules. Anal. Chem. 2010; 82:6224–6229. 20557026 10.1021/ac101041s PMC3172322

[4] Scott S. , Xu Z.M., Kouzine F., Berard D.J., Shaheen C., Gravel B., Saunders L., Hofkirchner A., Leroux C., Laurin J. et al. Visualizing structure-mediated interactions in supercoiled DNA molecules. Nucleic Acids Res. 2018; 46:4622–4631. 29684182 10.1093/nar/gky266 PMC5961182

[5] Scott S. , Shaheen C., McGuinness B., Metera K., Kouzine F., Levens D., Benham C.J., Leslie S. Single-molecule visualization of the effects of ionic strength and crowding on structure-mediated interactions in supercoiled DNA molecules. Nucleic Acids Res. 2019; 47:6360–6368. 31106378 10.1093/nar/gkz408 PMC6614806

[6] Adamcik J. , Jeon J.-H., Karczewski K.J., Metzler R., Dietler G. Quantifying supercoiling-induced denaturation bubbles in DNA. Soft Matter. 2012; 8:8651–8658.

[7] Irobalieva R.N. , Fogg J.M., Catanese D.J. Jr., Sutthibutpong T., Chen M., Barker A.K., Ludtke S.J., Harris S.A., Schmid M.F., Chiu W. et al. Structural diversity of supercoiled DNA. Nat. Commun. 2015; 6:8440. 26455586 10.1038/ncomms9440 PMC4608029

[8] Kouzine F. , Wojtowicz D., Baranello L., Yamane A., Nelson S., Resch W., Kieffer-Kwon K.R., Benham C.J., Casellas R., Przytycka T.M. et al. Permanganate/S1 Nuclease Footprinting Reveals Non-B DNA Structures with Regulatory Potential across a Mammalian Genome. Cell Syst. 2017; 4:344–356. 28237796 10.1016/j.cels.2017.01.013 PMC7432990

[9] Aboul-ela F. , Bowater R., Lilley D. Competing BZ and helix-coil conformational transitions in supercoiled plasmid DNA. J. Biol. Chem. 1992; 267:1776–1785. 1730717

[10] Oberstrass F.C. , Fernandes L.E., Bryant Z. Torque measurements reveal sequence-specific cooperative transitions in supercoiled DNA. Proc. Natl. Acad. Sci. USA. 2012; 109:6106–6111. 22474350 10.1073/pnas.1113532109 PMC3341030

[11] Nelson P. Transport of torsional stress in DNA. Natl. Acad. Sci. USA. 1999; 25:14342–14347. 10.1073/pnas.96.25.14342 PMC24438 10588707

[12] Kowalski D. , Natale D.A., Eddy M.J. Stable DNA unwinding, not ‘breathing,’ accounts for single-strand-specific nuclease hypersensitivity of specific A+ T-rich sequences. Proc. Nati. Acad. Sci. 1988; 5:9464–9468. 10.1073/pnas.85.24.9464 PMC282773 2849106

[13] Mizuuchi K. , Mizuuchi M., Gellert M. Cruciform structures in palindromic DNA are favored by DNA supercoiling. J. Mol. Biol. 1982; 156:229–243. 6283098 10.1016/0022-2836(82)90325-4

[14] Singleton C.K. , Klysik J., Stirdivant S.M., Wells R.D. Left-handed Z-DNA is induced by supercoiling in physiological ionic conditions. Nature. 1982; 299:312. 6287292 10.1038/299312a0

[15] Zhabinskaya D. , Benham C.J. Theoretical analysis of competing conformational transitions in superhelical DNA. PLoS Comput. Biol. 2012; 8:e1002484. 22570598 10.1371/journal.pcbi.1002484 PMC3343103

[16] Zhabinskaya D. , Madden S., Benham C.J. SIST: stress-induced structural transitions in superhelical DNA. Bioinformatics. 2015; 31:421–422. 25282644 10.1093/bioinformatics/btu657

[17] Kouzine F. , Liu J., Sanford S., Chung H.-J., Levens D. The dynamic response of upstream DNA to transcription-generated torsional stress. Nat. Struct. Mol. Bio. 2004; 11:1092. 15502847 10.1038/nsmb848

[18] Levens D. , Baranello L., Kouzine F. Controlling gene expression by DNA mechanics: emerging insights and challenges. Biophys. Rev. 2016; 8:259–268. 28510225 10.1007/s12551-016-0216-8 PMC5425794

[19] Martis B.S. , Forquet R., Reverchon S., Nasser W., Meyer S. DNA supercoiling: An ancestral regulator of gene expression in pathogenic bacteria?. Comp. Struct. Biotechnol. J. 2019; 17:1047–1055. 10.1016/j.csbj.2019.07.013 PMC6700405 31452857

[20] Liu H. , Mulholland N., Fu H., Zhao K. Cooperative activity of BRG1 and Z-DNA formation in chromatin remodeling. Mol. Cell. Biol. 2006; 26:2550–2559. 16537901 10.1128/MCB.26.7.2550-2559.2006 PMC1430323

[21] Corless S. , Gilbert N. Effects of DNA supercoiling on chromatin architecture. Biophys. Rev. 2016; 8:245–258. 27738453 10.1007/s12551-016-0210-1 PMC5039215

[22] El Houdaigui B. , Forquet R., Hindré T., Schneider D., Nasser W., Reverchon S., Meyer S. Bacterial genome architecture shapes global transcriptional regulation by DNA supercoiling. Nucleic Acids Res. 2019; 47:5648–5657. 31216038 10.1093/nar/gkz300 PMC6582348

[23] Peter B.J. , Arsuaga J., Breier A.M., Khodursky A.B., Brown P.O., Cozzarelli N.R. Genomic transcriptional response to loss of chromosomal supercoiling in Escherichia coli. Genome Biol. 2004; 5:R87. 15535863 10.1186/gb-2004-5-11-r87 PMC545778

[24] Hatfield W. , Benham C. DNA topology-mediated control of global gene expression in Escherichia coli. Ann. Rev. Genet. 2002; 36:175–203. 12429691 10.1146/annurev.genet.36.032902.111815

[25] Yanisch-Perron C. , Vieira J., Messing J., Chambers S., Prior S., Barstow D., Minton N., Gilbert W. Improved M13 phage cloning vectors and host strains: nucleotide. Gene. 1985; 33:103–119. 2985470 10.1016/0378-1119(85)90120-9

[26] Keller W. Determination of the number of superhelical turns in simian virus 40 DNA by gel electrophoresis. Proc. Nati. Acad. Sci. 1975; 72:4876–4880. 10.1073/pnas.72.12.4876 PMC388835 174079

[27] Berard D. , Shayegan M., Michaud F., Henkin G., Scott S., Leslie S. Formatting and ligating biopolymers using adjustable nanoconfinement. Applied Physics Letters. 2016; 109:033702.

[28] Sasse-Dwight S. , Gralla J.D. KMnO4 as a probe for lac promoter DNA melting and mechanism in vivo. J. Biol. Chem. 1989; 264:8074–8081. 2722774

[29] Ha S. , Lowenhaupt K., Rich A., Kim Y., Kim K. Crystal structure of a junction between B-DNA and Z-DNA reveals two extruded bases. Nature. 2005; 7062:1183–1186. 10.1038/nature04088 16237447

[30] King G.A. , Gross P., Bockelmann U., Modesti M., Wuite G.J., Peterman E.J. Revealing the competition between peeled ssDNA, melting bubbles, and S-DNA during DNA overstretching using fluorescence microscopy. Proc. Nati. Acad. Sci. 2013; 110:3859–3864. 10.1073/pnas.1213676110 PMC3593895 23431161

[31] Zhang X. , Chen H., Le S., Rouzina I., Doyle P.S., Yan J. Revealing the competition between peeled ssDNA, melting bubbles, and S-DNA during DNA overstretching by single-molecule calorimetry. Proc. Nati. Acad. Sci. 2013; 110:3865–3870. 10.1073/pnas.1213740110 PMC3593865 23431154

[32] Bird A. DNA methylation patterns and epigenetic memory. Genes Dev. 2002; 16:6–21. 11782440 10.1101/gad.947102

[33] Michelotti E.F. , Sanford S., Levens D. Marking of active genes on mitotic chromosomes. Nature. 1997; 388:895–899. 9278053 10.1038/42282

[34] Palozola K.C. , Lerner J., Zaret K.S. A changing paradigm of transcriptional memory propagation through mitosis. Nat. Rev. Mol. Cell Biol. 2019; 20:55–64. 30420736 10.1038/s41580-018-0077-z PMC6557398

[35] Crozat E. , Philippe N., Lenski R.E., Geiselmann J., Schneider D. Long-term experimental evolution in Escherichia coli. XII. DNA topology as a key target of selection. Genetics. 2005; 169:523–532. 15489515 10.1534/genetics.104.035717 PMC1449116

[36] Fye R.M. , Benham C.J. Exact method for numerically analyzing a model of local denaturation in superhelically stressed DNA. Phys. Rev. E. 1999; 59:3408.

[37] Sershen C.L. A Dynamic Model of Superhelical DNA Denaturation. J. Appl. Math. Comput. 2020; 4:43–56.

