# Supplementary material for: Non-equilibrium structural dynamics of supercoiled DNA plasmids exhibits asymmetrical relaxation
Source: Nucleic Acids Res. 2022 Feb 21;50(5):2754–64. doi: 10.1093/nar/gkac101 (PMC8934633; doi:10.1093/nar/gkac101)
Supplement: gkac101_Supplemental_File [file gkac101_supplemental_file.pdf]

Supplementary Information (SI): Non-equilibrium structural dynamics of supercoiled DNA plasmids exhibit asymmetrical relaxation

Cynthia Shaheen[1,2,3], Cameron Hastie[1,2,3], Kimberly Metera[1], Shane Scott[1,4], Zhi Zhang[1], Sitong Chen[1], Gracia Gu[1], Lisa Weber[5], Brian Munsky[5], Fedor Kouzine[6], David Levens[6], Craig Benham[7], Sabrina R. Leslie[1,2,3]

[1] Department of Physics, McGill University, Montreal, QC, Canada H3A 2T8, [2] Michael Smith Laboratories, University of British Columbia, BC, Canada, V6T 1Z4, [3] Department of Physics and Astronomy, University of British Columbia, BC, Canada, V6T 1Z1, [4] Institute of Materials Science, Kiel University, 24142 Kiel, Germany, [5] Department of Chemical and Biological Engineering and School of Biomedical Engineering, Colorado State University, Fort Collins, CO 80523, USA, [6] Center for Cancer Research, National Cancer Institute, Bethesda, MD 20892, USA, and [7] Genome Center, University of California Davis, Davis, CA 95616, USA

1 Methods

1.1 DNA plasmids

Two plasmids were used for these experiments. The first, pUC19, is a common cloning vector. It is 2686 bp long and contains two potential supercoil-induced unwinding sites, one associated with the origin of replication and the second associated with the ampicillin resistance gene promoter region. The second plasmid, pFLIP-FUSE, which is 2498 bp long, has been described previously [6]. It contains the FUSE region of the *c-myc* oncogene. Since pFLIP-FUSE has the kanamycin resistance gene, the FUSE region is the only predicted supercoil-induced unwinding region at physiological conditions.

1.2 Oligonucleotides

All oligonucleotides were ordered from Integrated DNA Technologies (IDT). The oligonucleotide used for all pFLIP-FUSE experiments had Cy-3b conjugated via an NHS ester reaction to an amine modified carbon at the 5' end, and had the following sequence:

5' - /5AmMC6/ATA GAT CAT TTC AGG GAG CA - 3'

The NHS ester reaction was done in house, with DNA purified via HPLC purification. This oligo is complementary to one end of the FUSE unwinding region, and extends into the area that unwinds first according to theoretical calculation and previous experiments performed by Kouzine *et al* [14, 6]. It was selected to minimize the probability of hairpin formation (minimum ΔG = 0.24 kcal/mol, at 25°C calculated from Integrated DNA Technologies' Oligo Analyzer [7]). The minimum ΔG associated with self-dimer formation is -4.62 kcal/mol (25°C, [7]), compared to the ΔG of the perfect complement (-35.51 kcal/mol).

A 20 base long oligo complementary to a sequence in pFLIP-FUSE approximately nine hundred base pairs away from the unwinding site was used for a control experiment detailed below (Figure S5). This oligo had the sequence:

5' - /5ATTO550N/ ATG CCT GCT TGC CGA ATA TC- 3'

This oligo was labelled with Atto-550 via an NH-ester modification by IDT, then purified with HPLC.

The oligo used for all pUC19 experiments was 30 bases long and had the following sequence:

5' – /5AmMC6/AAA TGA AGT TTT AAA TCA ATC TAA AGT ATA – 3'

This oligo is complementary to the centre of the pUC19 unwinding site, which is predicted to unwind first, and has been used in previous experiments by Scott *et al* [10, 9]. The oligo contained an amine modified carbon at the 5' end and was labelled with Cy-3b in house via an NH-ester reaction, then HPLC purified in house. It was selected to minimize the formation of hairpins and the minimum ΔG associated with hairpin formation is 0.63 kcal/mol at 25°C [7]. The minimum ΔG associated with self-dimer

formation is -8.74 kcal/mol [7], compared to the ΔG of the perfect complement (-47.24 kcal/mol).

Measurement of plasmid supercoiling

The average number of supercoils and the supercoiling density of each population of topoisomers was determined by running the sample on a 2% agarose gel in 2 M Tris, 1 M acetic acid, 50 mM EDTA (TAE) buffer for 40 h at 2 V/cm along with reference bands (see [5] for in depth explanation of method) in the presence of various concentrations of chloroquine diphosphate and ethidium bromide. Each sample has a Gaussian distribution of topoisomers, centred around the mean number of supercoils in the sample. Figure S1 shows one such gel. Lane 1 shows the test sample and other lanes show reference samples (numbers of supercoils in the reference samples are displayed above the gel). All samples on this gel are pFLIP-FUSE. Through counting bands from the closest lane in the reference sample, the number of supercoils of the sample was determined to be -15 $\pm$ 1.5 (σ = -0.062 $\pm$ 0.006). Due to the mechanism of the topoisomerase reaction, all samples have a Gaussian distribution of topoisomers. The intensity volume of each band in the Gaussian spread was determined by integrating the intensity of all pixels in detected bands (Image Lab software, Bio-Rad). These values were plotted against number of supercoils and fit with a Gaussian function in MATLAB. The spread reported with each mean linking number is the standard deviation from the fit.

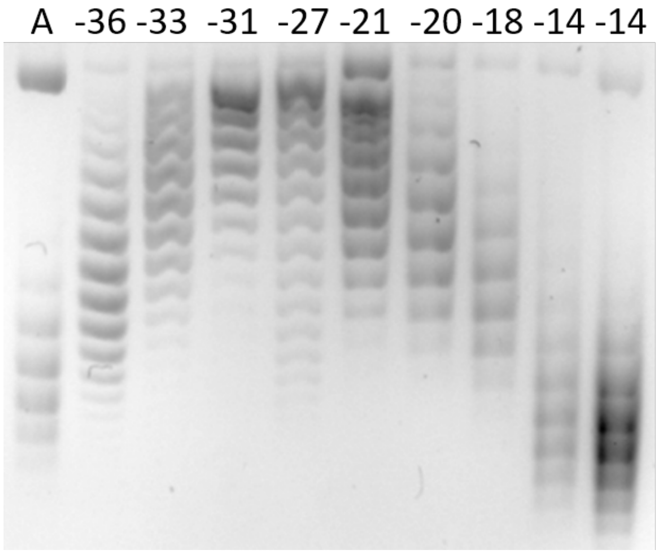

Figure S1: 2% agarose gel run in the presence of 6 mg/L of chloroquine diphosphate and 0.03 mg/L of ethidium bromide. Samples are different topoisomers of pFLIP-FUSE. Lane 1 (A) is the experimental sample being tested. Lanes 2 through 10 are a ladder of pFLIP-FUSE samples with different topoisomers. The number of supercoils of the brightest band in each lane is displayed above the lane. Each band away from that brightest band indicates the position of a discrete topoisomer. The intercalating agents positively supercoil the plasmids, such that plasmids with a true number of supercoils of -14 through -20 now run as though they had a positive linking number. The experimental sample has a number of supercoils of -15.

2 Analysis

2.1 Grid detection

Data analysis followed the methods outlined in Scott *et al* 2018 [10]. However, new methods were used for determining locations of the pits in the visible array. The array of pits was localized through a four step process: smoothing an image of the pits with a Weiner or band-pass filter, detecting edge features with a Canny edge detector, detecting spacing with a Hough transform, and detecting inclination with a Radon transform. The grids were detected separately on each raw video. First, the intensity of the frames of a video were averaged together, to give an image where filled pits were visible. Two new images were made by filtering the averaged image with either a Weiner filter with a 5 by 5 neighbourhood (MATLAB) or a Gaussian band pass filter using open source software by Crocker and Grier [1]. Canny edge detection is applied to both filtered images to detect edges of the circles that remain in the images after filtering. As extra edges are also detected, a Hough transform is used to detect circles in the images generated from the Canny edge detection. The magnitudes of the accumulator array peaks for each circle are used to determine which of the Weiner or band pass filtered image is used for the rest of the analysis. The Weiner filter works well in datasets with well-defined circles and high signal to noise. However, the Gaussian band pass filter out performs it in lower signal to noise experiments, at the cost of under-reporting visible pits. The pits detected through the Hough transform are averaged to create an ideal pit. The radius of the pit is calculated from here. This is correlated with the image for the Radon transform and intersections of the detected lines in the Radon transform are used to determine the final positions of the pits.

2.2 Exclusion of dark pits

Because the wide-field laser beam has a Gaussian beam profile, pits at the edges of the video were dark, leading to under-counting. To correct for this, we excluded the under-counted pits. We determined under-counting by comparing the count in each pit, summed over an entire data set (90 samplings), to the expected counts if the

particles were uniformly distributed, using a Chi-Squared test. We fit a two-dimensional Gaussian to the intensity profile of the videos and optimized a cutoff distance from the centre of the Gaussian, in order to optimize the Chi-Squared value, while maximizing statistics. We found that a cutoff of 0.55 sigma from the peak of the Gaussian was sufficient to eliminate under-counted pits. As the beam and pit positions were not constant between data sets, we scaled all results to be per 400 pits, the average number of pits in a full video.

2.3 Markov Chain Monte Carlo Sampler

Markov Chain Monte Carlo (MCMC) algorithms are methods for sampling probability density functions (PDFs), without the need for full analytic solutions of said PDFs [4]. In the following we present a Markov Chain Monte Carlo (MCMC) approach for extracting parameter posterior $\theta = [k_c, k_o, k_b, O_0]$ according to the system of nonlinear differential equations presented in the main article. Much of this work was done with guidance from [3, 12, 4, 8].

Within the context of Bayesian inference, the *posterior* PDF $P(\theta|D)$ can be described as the PDF of the model parameters θ with respect to some data D. Using Baye's theorem,

$$P(\theta|D) = \frac{1}{Z} P(D|\theta) P(\theta) \tag{1}$$

where $P(D|\theta)$ is the *likelihood*, $P(\theta)$ is the *prior*, and Z is a normalization constant which is notoriously difficult to calculate (*evidence*). To estimate parameters, MCMC algorithms compare ratios of the parameter posteriors, since the normalization constants cancel.

We employed a relatively simple formulation of MCMC known as the Metropolis algorithm in this study. This algorithm has two inputs: a cost function and a proposal function.

First, we need a function which compares positions in parameter space. Within the context of Eq.[1], this function refers to the product of the prior $P(\theta)$ and the likelihood $P(D|\theta)$. The prior in our system is uniform according to the principle of maximum entropy, since the only information we have is that the parameters are constrained to be within the interval $[a, b]$ [12]. Given the experimental data, the likelihood function was assumed to follow a multivariate Gaussian distribution that can be written as:

$$P(D|\theta) = C * \exp\left(-1/2(P - D)^T \Sigma^{-1} (P - D)\right) \tag{2}$$

where P is the model-predicted number of bound oligos per 100 pits, D is the observed number of bound oligos per 100 pits, Σ is the covariance matrix of the measurement errors, and C is a normalization constant. Because

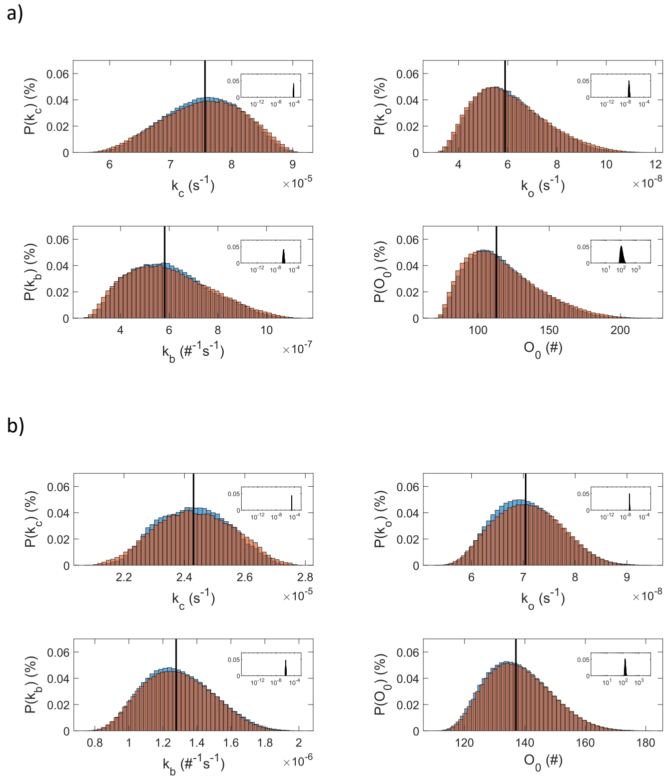

Figure S2: This figure shows the parameter posterior for pUC19 a) and pFLIP-FUSE b). The vertical line represents the mean of the PDF. The distributions are broken up into halves and are shown for each parameter. The significant overlap between each half acts as a qualitative measure of convergence. It must be noted that k_b and O_0 are displayed using units of absolute particle numbers per 400 pits while in table 1 of original text they are represented using molar concentration. Insets show the same distribution on a semi-log axis over the range corresponding to the uniformly distributed prior. This data was generated using $N \sim 1e9$ attempted MC steps with $[U_0, P_0] = [328, 9200]$ and $[U_0, P_0] = [328, 5200]$ for pUC19 and pFLIP-FUSE respectively.

each pit measurement is independent of the rest, we can assume that all measurement errors are independent and identically distributed with common variance σ^2, and this likelihood reduces to:

$$P(D|\theta) = C * \exp\left(-\frac{||P-D||}{2\sigma^2}\right). \quad (3)$$

The likelihood of the model given the parameters is the product of the prior with this likelihood and its logarithm can be written as:

$$\log(P(\theta|D)) = C' - \frac{||P-D||}{2\sigma^2} \quad (4)$$

where θ is within [a,b] and C' is again a normalization constant which does not depend on any of the model parameters. Here $||P - D||$ is the Euclidean distance between the predicted solution P and the data D, which is scaled by the variance of the data σ^2.

The predictions were numerically simulated given random parameters θ on the interval $[a, b]$, subject to the measured initial conditions. This was done using the standard MATLAB function ODE45 which is based on an explicit Runge-Kutta (4,5) formula, the Dormand-Prince pair [11, 2]. Given that a single set of parameters apply to multiple, related experimental curves (see Equation 2 in the original article), we used the cost of each predicted curve to simulate all related curves simultaneously.

Second, we need a reliable way of traversing parameter space. For this, a proposal PDF $Q(\theta'|\theta)$ is used to generate a *new* parameter sample θ' given an *old* sample θ. The proposal must satisfy a *detailed balance* condition, expressed mathematically as $Q(\theta'|\theta) = Q(\theta|\theta')$. This means that Q must be a symmetric distribution, and a simple choice is a Gaussian distribution $Q(\theta'|\theta) = N(\theta, \sigma_\theta)$, where σ_θ^2 is the variance in the step size for parameter θ.

Once these inputs have been specified, the algorithm can be implemented. Given some initial sample θ_i, the next sample θ_{i+1} is generated by the following scheme:

1. Generate a sample θ' using the proposal PDF $Q(\theta'|\theta_i)$.

2. Generate a random number R from a uniform distribution on the interval $[0, 1]$.

3. If $P_c(\theta')/P_c(\theta_i) > R$ then $\theta' \rightarrow \theta_{i+1}$. If not $\theta_i \rightarrow \theta_{i+1}$.

This is mathematically equivalent to a biased, random walk through parameter space. However, the random walk is biased only by the likelihood of the data with respect to a given set of parameter values, which in this case is related to the cost function $P_c(\theta)$. Care has been taken so that sampling remains unbiased by ensuring the prior PDF satisfies the principle of maximum entropy and by ensuring the proposal satisfies detailed balance.

The range of rates k_c, k_o, and k_b can be realistically bound to the interval $[10^{-12}, 10^1]$ (in units of h^{-1} or $h^{-1}counts^{-1}$ as applicable). This range was determined by solving the differential equations (see Equation 1 of the main text) analytically, with the assumption that k_c and k_o are much smaller than both k_b and are slow relative to the time scale of an experimental concentration curve. This assumption follows our previous work [10]. Under these assumptions, k_b and O_0 can be estimated by fitting the concentration curves with:

$$B(t) = O_0[1 - \exp(-(U_0 - O_0)k_b t)] \quad (5)$$

using nonlinear least squares methods. The concentration of unbound oligos U_0 is a measured initial condition. The concentrations of open plasmids O_0 obtained from this calculation for concentration curves $B(t)$ obtained using different incubation times were used along with equation 2 from the main paper to coarsely estimate k_o, and k_c. These estimates were used to determine a realistic interval for k_c, k_o, and k_b. The range of O_0 must be bound by $Z^+ \in [0, P_0]$.

Heuristically, the algorithm has converged if the joint distributions for an arbitrary division have identical features. This can be seen in Figure 2a), where the distributions from the first and second half of the walk are identical. Furthermore, the Gelman-Rubin diagnostic was calculated using the estimated parameter PDF results for both plasmids. The value of the diagnostic was extremely close to unity in either case, indicating convergence [8]. The first 1% of run time is dedicated to adjusting proposal distributions to ensure an acceptance ratio between 20-50% for each proposed move. Given this acceptance ratio, a simulation time of $N \sim 1e9$, and the parameter ranges listed above, we can expect to sample parameter space thoroughly.

The result of our Monte Carlo sampling is a joint posterior for all parameters. These PDFs are shown in Figure 2 with the parameter median (solid line). The exact statistics are shown in a table of the main article. Using the median and a range of the parameter choices (1000 random samples of the posterior PDF), we have constructed the corresponding fits shown in Figure 3. The data is well within the range of predictions shown using 1000 random samples of the parameter PDFs. However, this is not the case 3 hours after incubation for pFLIP-FUSE. This deviation is likely a result of our simplistic model.

It must also be noted that our parameters, specifically k_o, k_b, and O_0, were correlated (see Figure 4. However, the magnitude of these correlations diminishes with more curves at additional incubation times (see results for pFLIP-FUSE vs pUC19 in Figure 4).

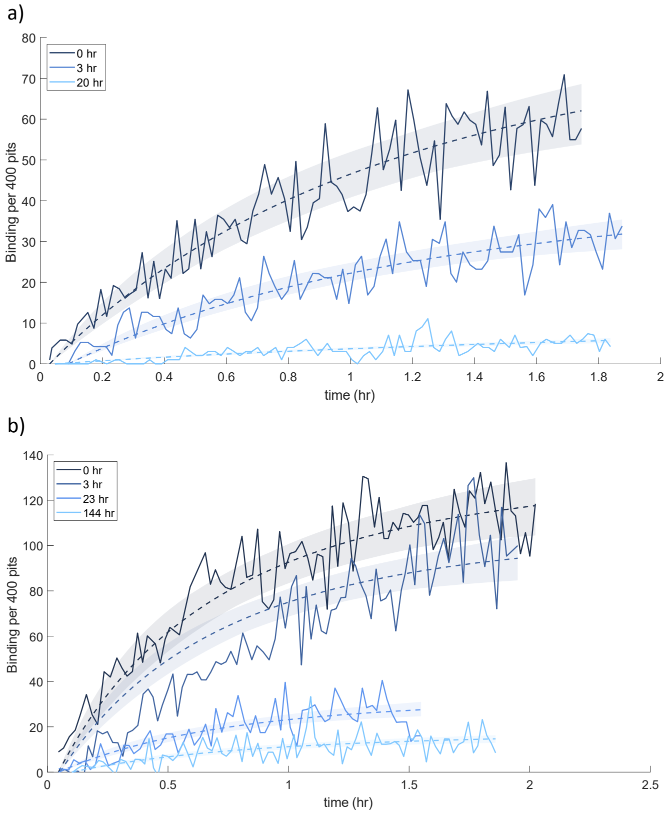

Figure S3: Raw data (solid) and the estimated fits (dashed) vs time for binding curves corresponding to the (a) pUC19 and (b) pFLIP-FUSE plasmids. Note that the data in the main article is shown using a running average. The incubation time before oligo introduction is shown in the legend. For pUC19, we have calculated fits for data taken 0, 3, and 20 hours after incubation, and for pFLIP-FUSE we have calculated fits for data taken 0, 3, 23, and 144 hours after incubation. The shaded region represents the spread in the fits using 1000 random samples of the parameter posteriors. This data was generated using $N \sim 1e9$ attempted MC steps with $[U_0, P_0] = [328, 9200]$ and $[U_0, P_0] = [328, 5200]$ for pUC19 and pFLIP-FUSE respectively.

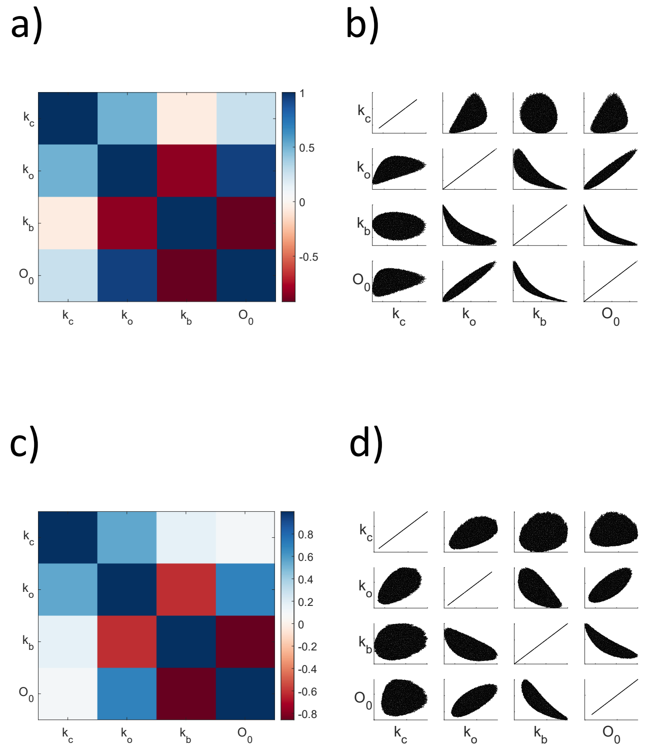

Figure S4: a)-b) The Pearson correlation between parameter PDFs and the relationship between parameter PDFs respectively for the pUC19 plasmid. c)-d) The Pearson correlation between parameter posterior and the relationship between parameter posterior for the pFLIP-FUSE plasmid.

3 Controls

3.1 Testing for mismatching after melting plasmids

When plasmids are heated and cooled rapidly, the two strands of DNA can mismatch during cooling, leading to stable open loops throughout the entirety of the plasmid. To test for large amounts of these loops in our sample, we designed an oligonucleotide complementary to a point on the pFLIP-FUSE plasmid other than the unwinding site (see oligo-design section above for sequence details). We repeated the heating trials (heat to 95°C for 1 min and cooled at a rate of 5°C per min until they reached 37°C) with this new oligo in place of the oligonucleotide complementary to the unwinding site (Figure S5). All else was the same between these trials (except the dye used on the oligo: Atto 550 as opposed to Cy3b). To compensate for the different brightnesses of the dye, dark pits, which could lead to ambiguity in the oligo count,

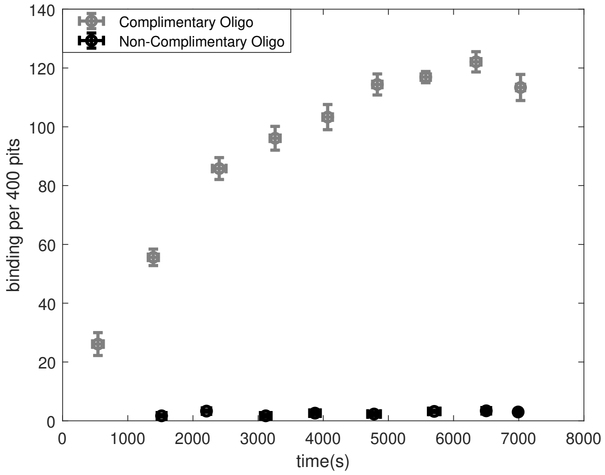

Figure S5: Control experiment to test for DNA mismatching in pFLIP-FUSE during cooling after heat treatment. Grey curve shows an oligo complementary to the unwinding site binding to plasmids that were heated to 95°C for 1 min and cooled at a rate of 5°C per min until they reached 37°C. The grey curve is the same curve as in Figure 2B (0 h incubation) of the main text. The black curve shows oligo-plasmid binding between the pFLIP-FUSE plasmid and an oligo complementary to a site away from the FUSE unwinding region.

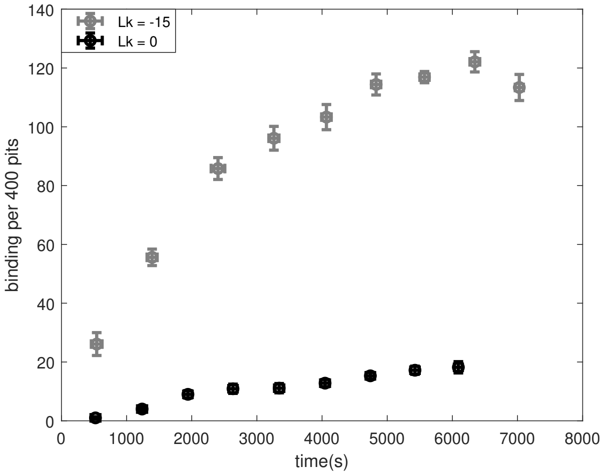

Figure S6: Oligo-plasmid interactions after heating and cooling a sample of fully relaxed pFLIP-FUSE plasmids (Lk=0) (dark curve). Plasmids were were heated to 95°C for 1 min and cooled at a rate of 5°C per min until they reached 37°C. The grey curve is the same curve as in Figure 2B (0 h incubation) in the main text for a direct comparison.

were excluded. All values are scaled to binding observed per 400 pits (one field of view). There was a small amount of binding observed with this oligo. After two hours of reacting with the plasmid, this binding was on the order of 10 bound complexes per 400 pits. This is in stark contrast to the 120 bound complexes observed per 400 pits when the oligo complementary to the unwinding site was used.

Additionally, if there was significant mismatching during cooling, since the mismatching would differ from plasmid to plasmid, we would expect smearing to appear on the gel after potassium permanganate finger printing analysis, which is not present. Instead, we see clear bands corresponding to ssDNA at only two predictable locations, supporting that the effect we see is due to the secondary structure dynamics of the unwinding site and not due to melting of the entire plasmid.

3.2 Supercoiling dependency of the heat perturbation

To check that this excess unwinding after heating is linked to supercoiling, we also tried heating and cooling a fully relaxed plasmid (Figure S6). Post cooling, the unwinding site in the uwound plasmid was still open in a small fraction of plasmids, leading to some binding (approximately 20 bound per 400 pits two hours after adding the oligo). However, there is a clear increase in binding with supercoiling density, suggesting that supercoiling still has an effect on how stably the unwinding site opens.

3.3 Oligo-plasmid interaction of pUC19 incubated at 37°C

There was no significant difference in the binding of oligos to supercoiled pUC19 when pUC19 was incubated at 37°C for various lengths of time prior to the addition of the oligo (Figure S7). This suggests that the plasmids have reached an equilibrium partition of wound and unwound plasmids quickly.

4 Potassium permanganate footprinting

4.1 Mapping

The locations of the secondary structures in pFLIP-FUSE were determined through running a potassium permanganate footprinting analysis with several different restriction enzymes (see main paper for full methods for the assay). Potassium permanganate was added to plasmids to oxidize any single stranded regions. The plasmids were cut with a restriction enzyme, then treated with S1 nuclease. Restriction enzymes used were PciI, ApoI, BstBI, and NciI-HF (all from New England Biolabs). From the differences in the lengths of the resulting fragments (Fig-

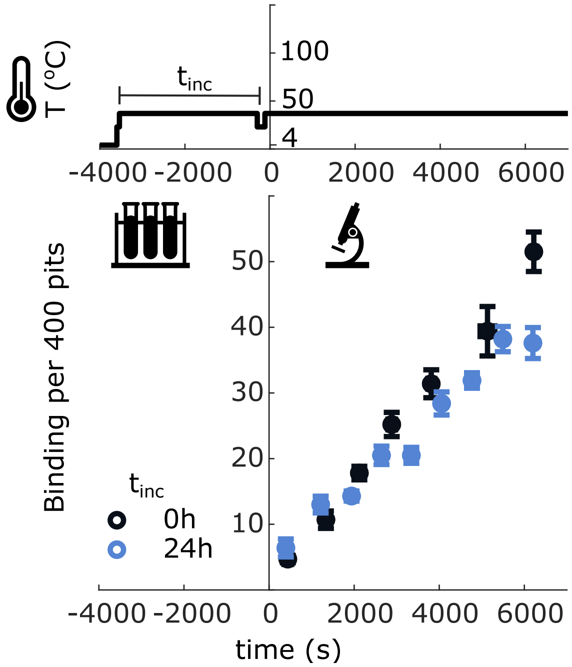

Figure S7: The number of fluorescent oligos bound to pUC19 (σ = -0.09) molecules per 400 pits, averaged over 10 videos, as a function of pre-experiment temperature treatment. pUC19 plasmids were increased from 4°C to 37°C and held at this temperature for either 0 h, 24 h prior to the addition of the oligo and oxygen scavengers at t = 0 s. All error bars represent the standard error of the mean. Top trace indicates the temperature of the plasmids. The decrease to room temperature just before time 0 indicates when the sample was transferred from the thermal cycler to the microscope.

ure S8), two cut sites were found in the plasmid. The first cut site corresponded to the expected location of the unwinding site. The second cut site corresponded to a location predicted to contain both a cruciform susceptible region and Z-DNA susceptible region. When the samples were heated and cooled, the amount of plasmid containing the secondary structure increased. As the probability of cruciform formation increases with temperature, while the probability of Z-DNA formation decreases with temperature (Figure S9 A), we hypothesize that this second structure is a cruciform.

Similarly, in pUC19, there were two cut sites, one corresponding to the primary unwinding site, and one corresponding to a region that contains both the secondary unwinding site and Z-DNA susceptible regions. As the amount of the second cut increased with temperature, and the probability of unwinding increased with temperature while the probability of Z-DNA formation decreased (Figure S9 B), we concluded that this second region was the secondary unwinding site.

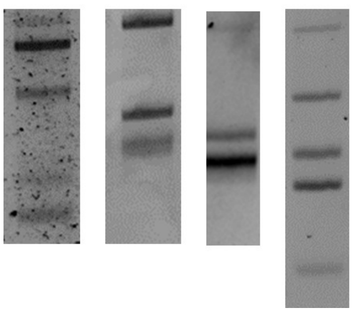

PciI	ApoI	BstBI	NcoI
2500	2500	2500	2500
2000	1300	1300	1500
1300	1100	1100	1100
600			850
400			500

Figure S8: Potassium permanganate footprinting gels showing pFLIP-FUSE after being cut by PciI, ApoI, BstBI, or NcoI-HF. Left is individual lanes of a 1% agarose gel run in TAE for 60 min at 120 V. Right is a table summarizing the approximate length of each band in the lanes on the left.

4.2 pFLIP-FUSE σ = -0.058 +/- 0.007

In the main paper, we presented the changes in secondary structures observed on potassium permanganate footprinting gels between supercoiled pFLIP-FUSE plasmids (σ = -0.104 +/- 0.010) that were chilled or heated before they were brought to 37°C. Here, we've repeated the experiment with plasmids supercoiled to a physiological level (σ = -0.058 +/- 0.0007). Fewer bands are visible here than in more highly supercoiled plasmids. Plasmids either contained no secondary structure (2500 bp band), just an open unwinding site (1500 bp and 850 bp bands) or just a cruciform (2000 bp band, the 500 bp band it pairs with was too dim to observe). Though the 2000 bp band was dim, it was present, unlike as observed at higher numbers of supercoils. Additionally, the band at 1000 bp is not present, though may be incredibly faint, suggesting a lack of plasmids that simultaneously contained both an open unwinding region and open cruciform region.

4.3 Asymmetry in measured rates

We observed an asymmetry in the state transitions of plasmids that were heated vs plasmids that were chilled prior to incubation at 37°C. Figure S11 illustrates this discrepancy. Using the transition rates measured from the heated plasmid, we simulated the binding curve we would expect to observe in an experiment where plasmids that started 100 % in the closed state were incubated at 37°C for various lengths of time before the addition of the oligo. For pFLIP-FUSE, a greater change in binding as a function of incubation time was predicted with these simulations than is observed experimentally. For pUC19, the experimental plasmid reached equilibrium far sooner than predicted by the measured rates.

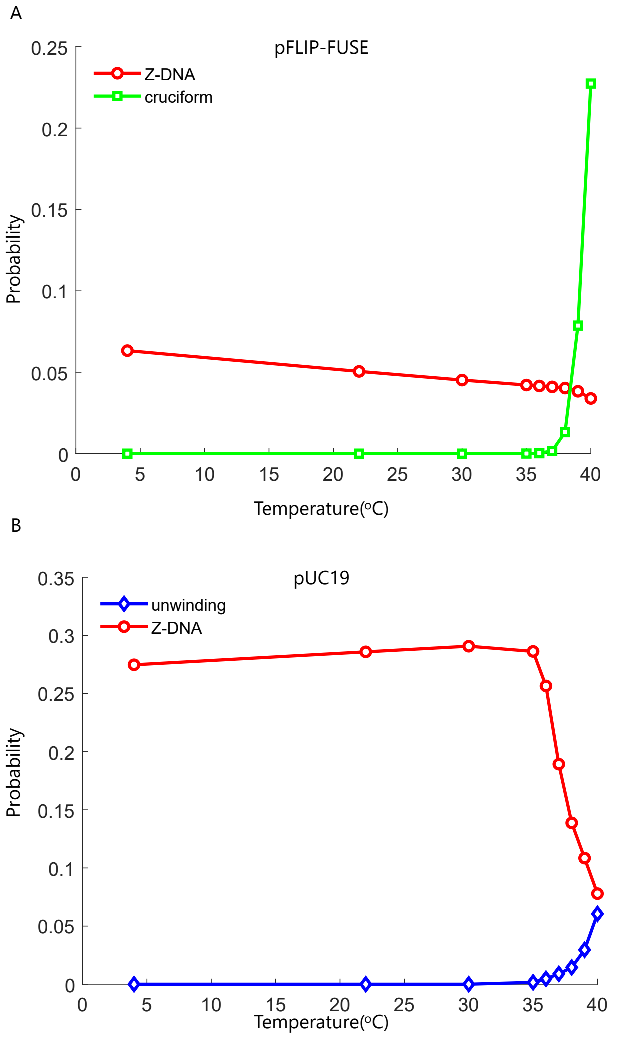

Figure S9: Probability of secondary structures occurring around the detected cut locations in **A** pFLIP-FUSE σ = -0.104 and **B** pUC19 σ = -0.0968 as a function of temperature, according to the DZCB-*trans* model [14]. All calculations were run at a monovalent ionic strength of 22.5 mM. Red circles represent Z-DNA, blue diamonds represent unwinding, and green squares represent cruciforms. The two most probable sites in each cut region were graphed.

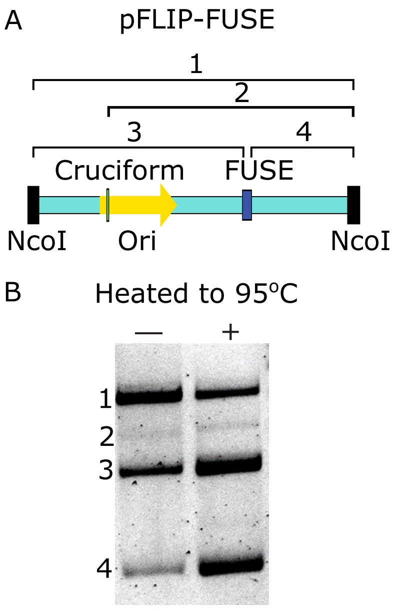

Figure S10: Potassium permanganate footprinting analysis of pFLIP-FUSE σ = -0.058 +/- 0.007 cut with NcoI-HF and S1 nuclease. All buffer conditions and heat treatments are the same as in Figure 4 in the main paper. **A** Mapping of the observed bands to cut sites on the plasmid. **B** Left lane, samples were heated from 4°C to 37°C prior to the addition of potassium permanganate. **B** Right lane, samples were heated to 95°C for 1 min and cooled step-wise at a rate of 5°C/min until they reached 37°C, when the potassium permanganate was added.

4.4 Predicted structures in pFLIP-FUSE and pUC19

Figure S12 shows the predicted probabilities of formation of the structures detected through permanganate footprinting (see Mapping subsection above). The detected structures were the FUSE unwinding region and a cruciform susceptible region. We predict a competition in the energetics of these structures. Both structures are predicted to increase in probability with increasing temperature (Figure S12 B and C). In more relaxed molecules (σ closer to 0) the cruciform is favoured over the unwinding site (Figure S12 A). As the plasmids become more supercoiled, the unwinding site becomes favoured. As the unwinding probability increases, the cruciform probability decreases, until there is sufficient energy for both the cruciform and unwinding to occur, resulting in a second peak in the cruciform probability. This reflects the results observed in the footprinting gels. At σ = -0.058 $\pm$ 0.007, we observed a band corresponding to plasmids that had an open cruciform and no open unwinding region, and no band corresponding to both the unwinding region and cruciform being present at the same time (Figure S10). At higher σ (-0.10 $\pm$ 0.01), we observed the opposite,

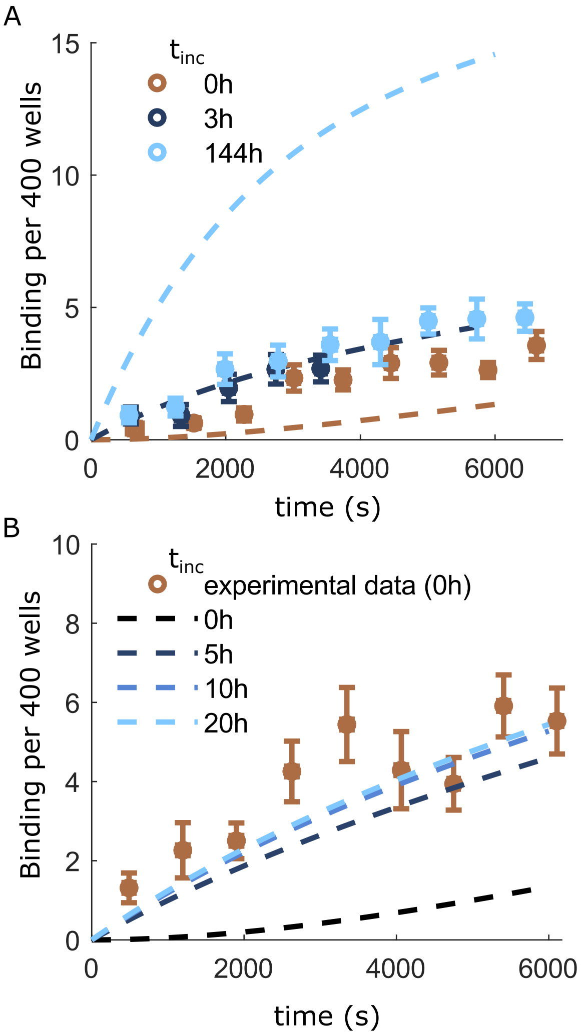

Figure S11: Comparison of measured binding curves of chilled plasmids to the predicted amount of binding given the kinetic rates measured from heated plasmids (reported in Table 1 in the main text). **A** *Circles*: pFLIP-FUSE oligonucleotide binding over time. pFLIP-FUSE samples (σ = -0.062 ± 0.006) were heated to 37°C for various lengths of time after storage at 4°C before the addition of the complementary oligo. This is the data reported in Figure 2A of the main text. *Dashed lines*: The predicted binding curves using the kinetic rates reported in Table 1 in the main text, assuming that time = 0 h had 0 unwound plasmids. The colour of the curves and circles correspond to the incubation times used. **B** *Circles* pUC19 (σ = -0.054 ± 0.006) oligo binding over time of plasmids heated to 37°C from 4°C and immediately mixed with the oligo. This is the data reported as the 'control' curve in Figure 3 of the main text. *Dashed lines*: The predicted binding curves using the kinetic rates reported in Table 1 in the main text at a series of incubation times, assuming that time = 0 h had 0 unwound plasmids. The discrepancies in the predicted and experimental curves illustrates an asymmetry in the transition rates dependent on the history of the plasmid prior to the addition of the oligo.

a band corresponding to both the unwinding region and cruciform being present at once, but no band corresponding to just the cruciform being present (Figure 4A from the main text). This matches the qualitative trend we predicted, though the transition from the unwinding site and cruciform forming separately to them forming on the same plasmids occurred at a lower superhelicity than predicted.

The behaviour of the two unwinding sites in pUC19 are also predicted to exhibit a competition between them. As plasmids become more negatively supercoiled, the probability of the more probable site unwinding (site 1, the focus of this work) increased. However, at particularly high supercoiling, site 2 (upstream of the ampicillin resistance gene) is predicted to open as well, and the probability of site 1 opening actually decreases as site 2 now absorbs some of the available energy (Figure S13 A). As temperature increases, the probability of both sites unwinding increases, with site 1 always unwinding at lower temperatures than site 2 (Figure S13 B-C). At high temperatures, the combined probability of each site opening can exceed 1, suggesting that at these conditions, plasmids that contain unwound sites at both conditions are predicted. This reflects what was observed in the footprinting analysis (Figure 4 of the main text). Plasmids that were approaching 37°C from a lower temperature had a larger population of plasmids with just site 2 open than plasmids with both site 1 and 2 open. However, plasmids approaching 37°C from a high temperature had a higher population of plasmids with both sites open than just site 2.

4.5 Ordering of structural transitions in pFLIP-FUSE

We conducted the footprinting assay on pFLIP-FUSE (σ= -0.050 ± 0.007) at a series of temperatures and concentrations of sodium chloride (Figure S14). We found that at low temperatures (0°C or 22.5°C with 10 mM NaCl), no secondary structures were detected (Figure S14 B, lanes 1 and 2). Likewise, almost no secondary structures were detected at high salt concentrations (100 mM NaCl at 37°C) (Figure S14 C, lane 2). However, as we increased temperature or decreased salt concentration, we observed that the FUSE unwinding region opens, leading to a second cut site in the oxidized plasmids (Figure S14 B, lanes 3 and 4, and C, lane 1). In plasmids that were heated to 95°C before being brought to 37°C, we observed a faint band corresponding to the potential cruciform discussed in the main text. The heated sample was treated with potassium permanganate within 5 minutes of cooling back to 37°C. All other samples were incubated at the experimental temperature in the experimental buffer for approximately 30 min before potassium permanganate treatment.

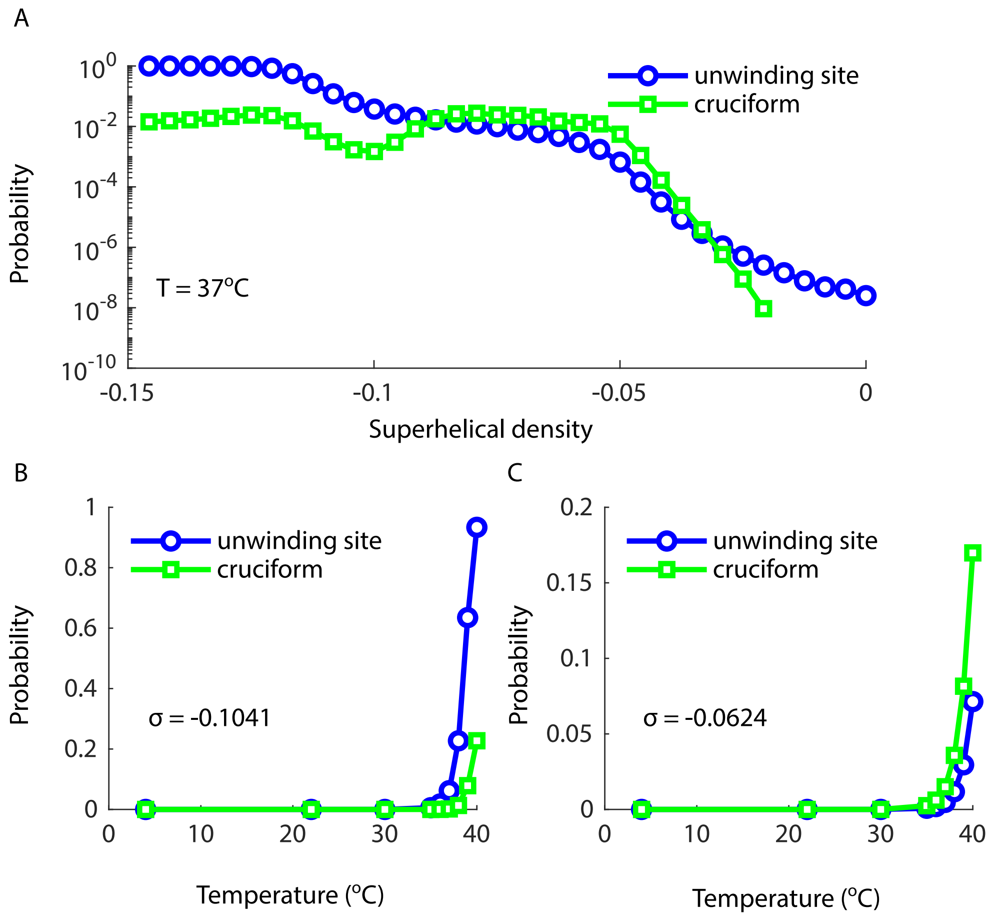

Figure S12: Theoretical probabilities of detected structural transitions in pFLIP-FUSE using the DZCB-*trans* algorithm [13]. The structures detected through footprinting analysis were the unwinding region and a cruciform. The maximum probability of each of these regions was calculated at each condition reported. All calculations were done at 22.5 mM salt. **A** The probability of cruciform formation and unwinding at 37°C as a function of superhelicity. A log scale was used to better illustrate the low probability behaviours. **B-C** The probability of cruciform formation and unwinding at σ = -0.1041 (**B**) and σ = -0.0624 (**C**) as a function of temperature.

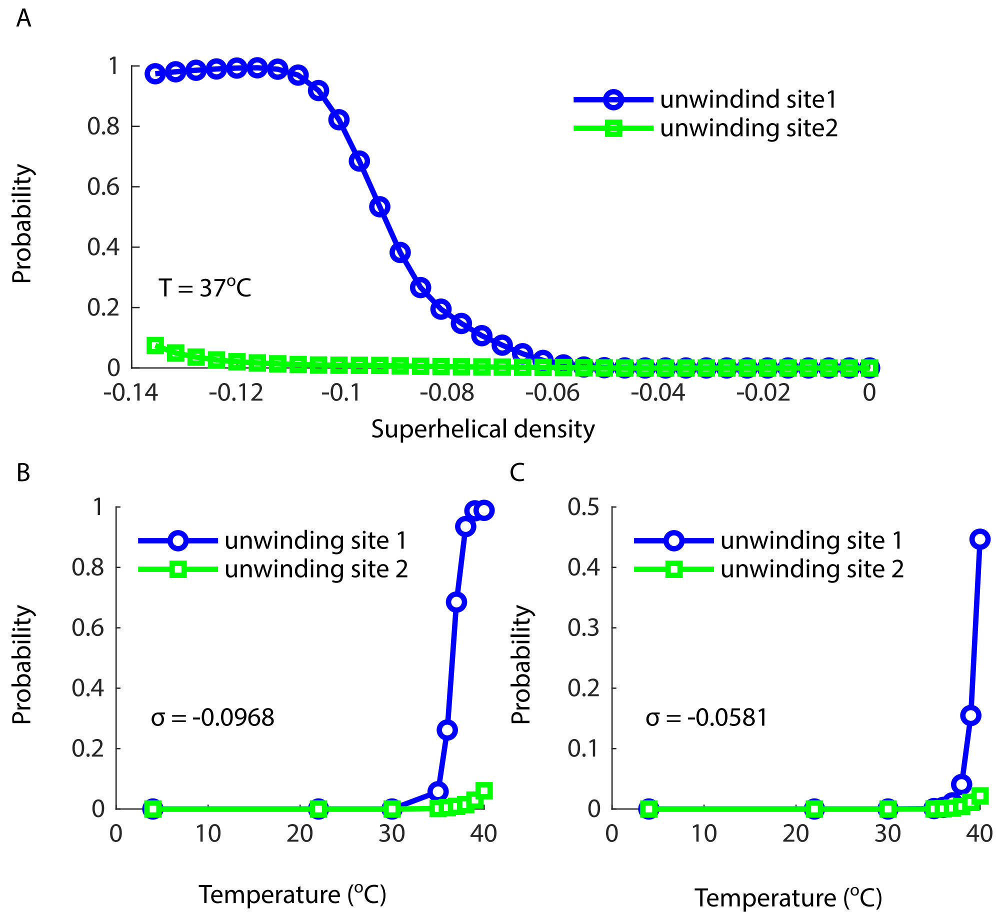

Figure S13: Theoretical probabilities of detected structural transitions in pUC19 using the DZCB-*trans* algorithm [13]. The structures detected through footprinting analysis were two unwinding regions. Site 1 is the region focused on in this study, while site 2 is a region located upstream of the ampicillan resistance gene. The maximum probability of each of these regions was calculated at each condition reported. All calculations were done at 22.5 mM salt. **A** The probability of unwinding at each site at 37°C as a function of superhelicity. **B-C** The probability of unwinding at each site at σ = -0.0968 (**B**) and σ = -0.0581 (**C**) as a function of temperature.

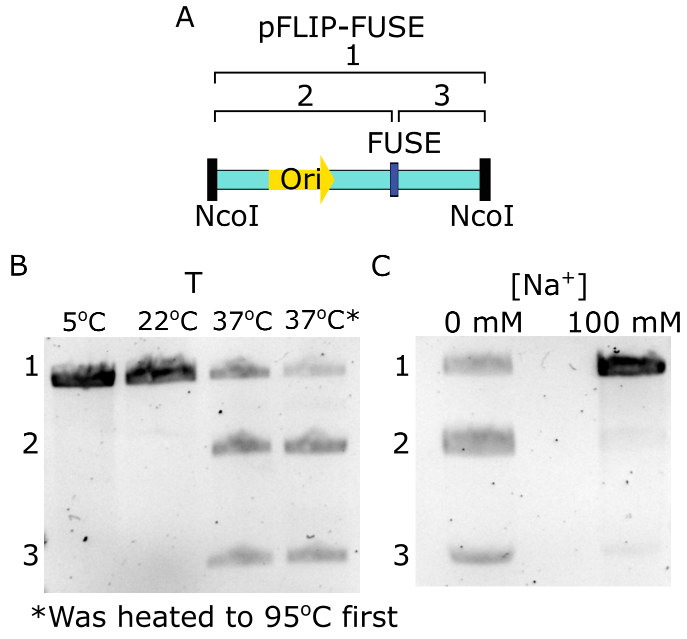

Figure S14: Potassium permanganate fingerprinting of pFLIP-FUSE plasmid σ= -0.050 ± 0.007. Plasmids were oxidized with $KMnO_4$, cut with NCoI-HF (NEB), then cut with S1 nuclease. **A** A map of the observed cut location and resulting fragments of pFLIP-FUSE. **B** Resulting fragments of pFLIP-FUSE oxidized at 5°C, 22°C, 37°C, and 37°C immediately after heating to 95°C and cooling as described in the main text. All other plasmids were held at the indicated temperature for 30 min prior to oxidization. All samples were in a buffer containing 20 mM tris (pH 7.97) and 10 mM NaCl. **C** Resulting fragments of pFLIP-FUSE oxidized in buffer containing 20 mM tris (pH 7.97) and 0 mM or 100 mM NaCl. Samples were treated at 37°C and were held at this temperature for 30 min prior to oxidization.

References

References

[1] J. C. Crocker and D. Grier. Matlab locating and tracking code.

[2] J. R. Dormand and P. J. Prince. A family of embedded runge-kutta formulae. *Journal of computational and applied mathematics*, 6(1):19–26, 1980.

[3] D. Frenkel and B. Smit. *Understanding molecular simulation: from algorithms to applications*, volume 1. Elsevier, 2001.

[4] D. W. Hogg and D. Foreman-Mackey. Data analysis recipes: Using markov chain monte carlo. *The Astrophysical Journal Supplement Series*, 236(1):11, 2018.

[5] W. Keller. Determination of the number of superhelical turns in simian virus 40 dna by gel electrophoresis. *Proceedings of the National Academy of Sciences*, 72(12):4876–4880, 1975.

[6] F. Kouzine, J. Liu, S. Sanford, H.-J. Chung, and D. Levens. The dynamic response of upstream dna to transcription-generated torsional stress. *Nature structural molecular biology*, 11(11):1092, 2004.

[7] R. Owczarzy, A. V. Tataurov, Y. Wu, J. A. Manthey, K. A. McQuisten, H. G. Almabrazi, K. F. Pedersen, Y. Lin, J. Garretson, N. O. McEntaggart, et al. Idt scitools: a suite for analysis and design of nucleic acid oligomers. *Nucleic acids research*, 36(suppl_2):W163–W169, 2008.

[8] V. Roy. Convergence diagnostics for markov chain monte carlo. *Annual Review of Statistics and Its Application*, 7:387–412, 2020.

[9] S. Scott, C. Shaheen, B. McGuinness, K. Metera, F. Kouzine, D. Levens, C. J. Benham, and S. Leslie. Single-molecule visualization of the effects of ionic strength and crowding on structure-mediated interactions in supercoiled dna molecules. *Nucleic Acids Res*, 47(12):6360–6368, 2019.

[10] S. Scott, Z. M. Xu, F. Kouzine, D. J. Berard, C. Shaheen, B. Gravel, L. Saunders, A. Hofkirchner, C. Leroux, J. Laurin, D. Levens, C. J. Benham, and S. R. Leslie. Visualizing structure-mediated interactions in supercoiled dna molecules. *Nucleic Acids Res*, 46(9):4622–4631, 2018.

[11] L. F. Shampine and M. W. Reichelt. The matlab ode suite. *SIAM journal on scientific computing*, 18(1):1–22, 1997.

[12] U. Von Toussaint. Bayesian inference in physics. *Reviews of Modern Physics*, 83(3):943, 2011.

[13] D. Zhabinskaya and C. J. Benham. Theoretical analysis of competing conformational transitions in superhelical dna. *PLoS computational biology*, 8(4):e1002484, 2012.

[14] D. Zhabinskaya, S. Madden, and C. J. Benham. Sist: stress-induced structural transitions in superhelical dna. *Bioinformatics*, 31(3):421–422, 2015.
